# RPA complexes in *Caenorhabditis elegans* meiosis; unique roles in replication, meiotic recombination and apoptosis

**DOI:** 10.1093/nar/gkaa1293

**Published:** 2021-01-21

**Authors:** Adam Hefel, Masayoshi Honda, Nicholas Cronin, Kailey Harrell, Pooja Patel, Maria Spies, Sarit Smolikove

**Affiliations:** Department of Biology, The University of Iowa, Iowa City, IA 52242, USA; Department of Biochemistry, The University of Iowa Carver College of Medicine, Iowa City, IA 52242, USA; Department of Biochemistry, The University of Iowa Carver College of Medicine, Iowa City, IA 52242, USA; Department of Biology, The University of Iowa, Iowa City, IA 52242, USA; Department of Biology, The University of Iowa, Iowa City, IA 52242, USA; Department of Biochemistry, The University of Iowa Carver College of Medicine, Iowa City, IA 52242, USA; Department of Biology, The University of Iowa, Iowa City, IA 52242, USA

## Abstract

Replication Protein A (RPA) is a critical complex that acts in replication and promotes homologous recombination by allowing recombinase recruitment to processed DSB ends. Most organisms possess three RPA subunits (RPA1, RPA2, RPA3) that form a trimeric complex critical for viability. The *Caenorhabditis elegans* genome encodes RPA-1, RPA-2 and an RPA-2 paralog RPA-4. In our analysis, we determined that RPA-2 is critical for germline replication and normal repair of meiotic DSBs. Interestingly, RPA-1 but not RPA-2 is essential for somatic replication, in contrast to other organisms that require both subunits. Six different hetero- and homodimeric complexes containing permutations of RPA-1, RPA-2 and RPA-4 can be detected in whole animal extracts. Our *in vivo* studies indicate that RPA-1/4 dimer is less abundant in the nucleus and its formation is inhibited by RPA-2. While RPA-4 does not participate in replication or recombination, we find that RPA-4 inhibits RAD-51 filament formation and promotes apoptosis of a subset of damaged nuclei. Altogether these findings point to sub-functionalization and antagonistic roles of RPA complexes in *C. elegans*.

## INTRODUCTION

Replication protein A (RPA) is a heterotrimeric complex which binds single-stranded DNA (ssDNA) with high affinity [reviewed in (1,2)]. In most organisms, this complex consists of a large (70 kDa), medium (32 kDa) and small (14 kDa) subunit (RPA1, RPA2 and RPA3, respectively, in humans). Each subunit contains at least one oligonucleotide/oligosaccharide binding domain (OB fold), which give the complex its ssDNA binding activity (1). RPA removes secondary structures in ssDNA, a property which is critical for replication and recombination (3). RPA was originally isolated as a factor essential for human simian virus SV40 *in vivo* replication (4,5). The role of RPA in replication is not only determined by its ability to bind ssDNA, but also by indirect interaction with proteins that are part of the replication machinery, including PCNA (2,6) and pol α (7). RPA also plays a role in cell cycle signaling and the DNA damage response, where RPA promotes ATM activation, possibly through its interaction with the MRN complex (8), and indirect ATR activation (9,10). In humans, the DNA damage induced apoptotic response is stimulated by RPA2 hyperphosphorylation (11). Furthermore, double-strand DNA break (DSB) repair by homologous recombination (HR) also requires RPA, where its capacity to bind ssDNA and melt secondary structures is required for the assembly of the Rad51–ssDNA filament (2,12–14). RPA is also required for other forms of DNA repair where ssDNA is formed (15). The RPA complex is found in all eukaryotes, and its functions appear to be conserved.

RPA subunit composition is not limited to the three canonical subunits for all organisms; in some organisms paralogs are found. Subunit paralog identities vary between organisms, and have been driven by gene duplication events throughout evolution (16–18). The paralogs studied may retain the ancestral activities of the RPA subunit or lose some activities, but seldom neofunctionalise. For example, an RPA2 paralog RPA4, is found in several mammals. In humans, RPA4 shares some activities with RPA2 where both facilitate homologous recombination. However, RPA4 is unable to signal cell-cycle progression or support replication (19). Plants have multiple copies of RPA1, RPA2 and RPA3 subunits, an outcome of their evolutionary history that involves many genome duplications. For example, the *Arabidopsis* genome contains five RPA1-like subunits, two RPA2-like subunits, and two RPA3-like subunits (20). The different RPA1 paralogs in *Arabidopsis* diverged in their functions: atRPA1C promotes meiotic HR, whereas atRPA1B and atRPA1D act in DNA replication. Archaea have RPA compositions that differ from eukaryotes, where some are missing RPA3 and only possess a large RPA1-like subunit, and one example has an RPA1-like subunit which dimerizes (21–23).

Gamete formation requires the faithful execution of two main functions supported by RPA: replication and recombination. Germ cells replicate their genome and undergo mitotic divisions in their stem cell niche to produce cells that enter meiosis. These cells are then required to repair a multitude of programmed DSBs by the process of recombination to produce the crossovers necessary for the formation of viable gametes. Crossovers act as a physical tether between homologous chromosomes, allowing for proper segregation of these chromosomes at the end of meiosis I. In many organisms the absence of germline DSBs, or meiotic HR, leads to the formation of eggs and sperm that are inviable (24–27). In meiotic prophase I, DSBs form by the activity of the topoisomerase VI-like protein Spo11 [reviewed in (28)]. Spo11 breaks are resected by nucleases in an MRN(X)-dependent manner, leading to formation of ssDNA bound by RPA. To allow for strand invasion that leads to the formation of a double-Holliday junction, RPA is displaced by RAD51 [reviewed in (29)]. In the absence of RPA, *in vivo*, RAD51 cannot efficiently assemble a RAD51-ssDNA filament leading to an inability to repair DSBs via HR. When HR is impaired, DSBs can be repaired through other mechanisms, such as canonical non-homologous end-joining (cNHEJ) and alternative end-joining (alt-EJ) [reviewed in (30)]. In these repair events, ends are ligated together often in an error-prone way. However, they do not promote crossovers and thus do not promote proper meiotic chromosome segregation.

In *Caenorhabditis elegans*, there are orthologs for RPA1 (RPA-1) and RPA2 (RPA-2), as well as an additional subunit named RPA-4 which shares size and domain structure with RPA-2. It was previously shown that RPA-1 forms foci in pachytene nuclei (31–34). RPA-1 foci are formed concurrently with RAD-51 focus formation, but continue to accumulate when RAD-51 foci numbers start to diminish (34). These findings suggest that RPA-1 serves two roles in recombination: one essential for RAD-51 loading onto ssDNA and one in localization at crossover intermediates (34). RPA-1 localization increases following treatment with DNA damaging agents such as hydroxyurea (HU), UV, or ionizing radiation (IR), likely due to accumulation of ssDNA in these cells and the requirement for RPA in HR and the replication stress response (32,34). In a few studies, RPA-1 was shown to form a haze in mitotic germline nuclei (32,33,35,36). Knockdown of *rpa-1* by RNAi leads to embryonic lethality and defects in germline development (37,38). These studies are consistent with an essential role for RPA-1 in DNA replication. While RPA-1 has been thoroughly studied, the additional subunits of RPA found in *C. elegans* have not, raising questions about their functions.

Most germ cells of the *C. elegans* hermaphrodite germline undergo apoptosis leading to elimination of nuclei at the pachytene/diplotene transition. There are two known processes leading to germline apoptosis, one that is induced by stress (such as exogenously-induced DNA damage), and one that occurs as part of germline development termed ‘physiological apoptosis’ (39). Stress-induced apoptosis is considered to be a mechanism for removing nuclei that have a selective disadvantage, such as those that contain excessive or unrepairable DNA damage. It is generally accepted that physiological apoptosis is required for germline nuclei to act as nurse cells/nuclei, contributing their content to the oocytes that escape apoptosis (40). Mitogen-activated protein kinase (MAPK) signaling is required for licensing nuclei for apoptosis (40–42). MAPK acts on the core apoptotic machinery leading to activation of CEll Death abnormal-3 (CED-3, caspase) and CED-4 (Apaf-1), resulting in DNA fragmentation and the formation of a cell corpse (39). The corpses are then engulfed by CED-1-expressing somatic sheath cells to eliminate them from the germline (43). Germlines that are defective for apoptosis produce abnormal and smaller eggs (44). A more extreme phenotype is observed when MAPK signaling is blocked, leading to pachytene-like nuclei accumulation in the proximal gonad (45). In *C. elegans*, RPA was not shown to play a direct role in apoptosis. However, RPA is involved in DNA damage signaling through the recruitment of ATR (ATL-1), and it may play an indirect role in apoptosis signaling through its interaction with ssDNA (32).

Here, we determine the roles of RPA-2 and RPA-4 in *C. elegans* meiosis. Our results show that RPA-1 and RPA-2 are involved in germline DNA replication, while RPA-4 is not. RPA-2 and RPA-1 are also critical for DSB repair, where these proteins localize to programmed and exogenously induced DSBs, while RPA-4 plays a minor role in DSB repair. In contrast to other species, *C. elegans* RPA-2 is not completely required for RPA-1 focus formation, nor is it essential for somatic replication. Surprisingly, RPA-4 localizes to DSBs formed by replication defects and exogenous damage, and attenuates the loading of RAD-51. Additionally, *rpa-2; rpa-4* double mutants display an unusual germline progression defect, and we demonstrate that this phenotype is due to defects in apoptosis. Altogether our studies point to unique functions of distinct RPA complexes in *C. elegans*.

## MATERIALS AND METHODS

### Strains

Worms were maintained on NGM plates with lawns of OP50 *Escherichia coli* at 20°C. Strains used for experiments include N2 (Bristol), and contained the following alleles in the N2 genetic background:

SSM287 *rpa-2(ok1627) I; polq-1(tm2026) III /hT2 [bli-4(e937) let-?(q782) qIs48] (I;III)*
SSM288 *rpa-2(ok1627) I; cku-70(tm1524) III./hT2 [bli-4(e937) let-?(q782) qIs48] (I;III)*
SSM338 *rpa-2(ok1627) I; cku-70(tm1524) polq-1(tm202) III/hT2 [bli-4(e937) let-?(q782) qIs48] (I;III)*
SSM343 *rpa-4(iow21)I*
SSM346 *cku-70(tm1524); polq-1(tm202) III/hT2 [bli-4(e937) let-?(q782) qIs48] (I;III)*
SSM352 *rpa-2(ok1627) rpa-4(iow24) I/hT2 [bli-4(e937) let-?(q782) qIs48] (I;III)*
SSM354 *smIs34 [ced-1p::ced-1::GFP + rol-6(su1006)]; rad-51(iow53[GFP::rad-51]) spo-11(iow16) IV/nT1[qls 51] (IV, V)*
SSM387 *rpa-2(iow49[3xFLAG::rpa-2])I*
SSM389 *rpa-4(iow51)[3xFLAG::rpa-4])I*
SSM390 *rpa-4(iow21); rad-51(ok2218) IV/nT1[qIs51](IV;V)*.
SSM410 *rpa-2(ok1627) rpa-4(iow59[3xFLAG::rpa-4])I/hT2 [bli-4(e937) let-?(q782) qIs48] (I;III)*.
SSM473 *iowSi8[pie-1p::gfp1-10::him-3 3UTR + Cbr-unc119(+)] II rpa-1(iow89[gfp11::rpa-1])II; unc-119(ed3) III;*
SSM476 *rpa-1(iow92[OLLAS::rpa-1]) II*
SSM553 *rpa-4(iow51)[3xFLAG::rpa-4])I; rpa-1(iow92[OLLAS::rpa-1]) II; rpa-2(ok1627) I/hT2 [bli-4(e937) let-?(q782) qIs48] (I;III)*.
SSM554 *rpa-1(iow92[OLLAS::rpa-1]) II*; *rpa-4(iow24)* I; *rpa-2(ok1627)* I/hT2 [bli-4(e937) let-?(q782) qIs48] (I;III).
SSM555 *smIs34 [ced-1p::ced-1::GFP + rol-6(su1006)]; rpa-2(ok1627) I/hT2 [bli-4(e937) let-?(q782) qIs48] (I;III)*
SSM 556 *ollas::rpa-1II; rpa-2(ok1627) I/hT2 [bli-4(e937) let-?(q782) qIs48] (I;III)*.
SSM557 *rpa-4(iow51)[3xFLAG::rpa-4])I; spo-11 (ok79) IV / nT1 [qls51] (IV, V)*
SSM558 *rpa-4(iow21) I; rpa-1(iow92[OLLAS::rpa-1]) II*
SSM559 *rpa-2(iow49[3xFLAG::rpa-2])I*; *rpa-1(iow92[OLLAS::rpa-1]) II*
; *rpa-4(iow128[MYC::rpa-4) I*
SSM 560 *rpa-4(iow51)[3xFLAG::rpa-4])I; rpa-1(iow92[OLLAS::rpa-1]) II*
SSM561 *rpa-2(iow49[3xFLAG::rpa-2])I; rpa-1(iow92[OLLAS::rpa-1]) II*
SSM563 *rpa-2(iow127[3xFLAG::rpa-2])I; rpa-1(iow92[OLLAS::rpa-1]) II; rpa-4(iow21) I*
SSM 566 *rpa-4(iow51)[3xFLAG::rpa-4])I; smIs34 [ced-1p::ced-1::GFP + rol-6(su1006)]*
SSM567 *smIs34 [ced-1p::ced-1::GFP + rol-6(su1006)]; rpa-4(iow24)I; rpa-2(ok1627) I/hT2 [bli-4(e937) let-?(q782) qIs48] (I;III)*.
SSM569 *smIs34 [ced-1p::ced-1::GFP + rol-6(su1006)]; rpa-4(iow21)*
SSM570 *rpa-4(iow21) I; syp-2(ok307) V/nT1[unc-?(n754) let-?(m435)] (IV;V)*.
SSM577 *rpa-4(iow21) I; rad-51(iow53[GFP::rad-51]) spo-11(iow16) IV/nT1[qls 51] (IV, V)*
SSM594 *rpa-2(ok1627) I, ced-3(ok2734) IV*.
SSM596 *rpa-1(iow117)*/*mIn1[mIs14 dpy-10(e128)] II*

### Viability

L4 worms were singled onto NGM plates containing a small (1 cm^3^) lawn of OP50 *E. coli*. P0s were transferred to new plates twice a day for 4 days. Immediately after transfer eggs were counted, and adults were counted 4 days later.

### CRISPR

CRISPR/Cas9 was used to create the following strains for this publication:

SSM343 *rpa-4(iow21)I*
SSM352 *rpa-2(ok1627) rpa-4(iow24) I/hT2 [bli-4(e937) let-?(q782) qIs48] (I;III)*
SSM387 *rpa-2(iow49[3xFLAG::rpa-2])I*
SSM389 *rpa-4(iow51)[3xFLAG::rpa-4])I*
SSM410 *rpa-2(ok1627) rpa-4(iow59[3xFLAG::rpa-4])I/hT2 [bli-4(e937) let-?(q782) qIs48] (I;III)*.
SSM476 *rpa-1(iow92[OLLAS::rpa-1]) II*
SSM553 *rpa-4(iow51)[3xFLAG::rpa-4])I; rpa-1(iow92[OLLAS::rpa-1]) II; rpa-2(ok1627) I/hT2 [bli-4(e937) let-?(q782) qIs48] (I;III)*.
SSM559 *rpa-2(iow49[3xFLAG::rpa-2])I*; *rpa-1(iow92[OLLAS::rpa-1]) II*
; *rpa-4(iow128[MYC::rpa-4) I*
SSM561 *rpa-2(iow49[3xFLAG::rpa-2])I; rpa-1(iow92[OLLAS::rpa-1]) II*
SSM563 *rpa-2(iow127[3xFLAG::rpa-2])I; rpa-1(iow92[OLLAS::rpa-1]) II; rpa-4(iow21) I*
SSM 566 *rpa-4(iow51)[3xFLAG::rpa-4])I; smIs34 [ced-1p::ced-1::GFP + rol-6(su1006)]*
SSM596 *rpa-1(iow117)*/*mIn1[mIs14 dpy-10(e128)] II*

Micro-injection of 1-day-old adult worms was performed on 3% agarose pads, afterwards collected on a single NGM plate, and isolated to individual OP50 seeded plates the following morning. Plates were screened for the Rol or Dpy phenotypes generated by *dpy-10* point mutation introduced by co-CRISPR marker, adopted from (46). Wild-type F1s were isolated to individual plates for insertion screening by PCR and sequencing. tracrRNA, and crRNAs were obtained from IDT and mixed in the following concentrations: 14.35 μM Cas9-NLS (Berkeley MacroLab), 17.6 μM tracrRNA (IDT), 1.5 μM *dpy10* crRNA (IDT), 0.5 μM *dpy10* ssODN (IDT), 16.2 μM of target crRNA (IDT) and 6 μM of target ssODN (IDT). ssODNs and crRNAs used include the following:

**Table:** 

crRNA	
*3XFLAG::rpa-2*	UCCAGAAACUACUAACCAUGGUUUUAGAGCUAUGCU
*3XFLAG::rpa-4*	CGAAAUUUUGACACUAGCGAGUUUUAGAGCUAUGCU
*OLLAS::RPA-1*	UUUCAGAUAGUGAAAGAUGGGUUUUAGAGCUAUGCU
*rpa-4*	GUCGCCGUAUCCCUUACUGGUUUUAGAGCUAUGCU
	GAUGCAAGAGUCACUGGAAGUUUUAGAGCUAUGCU
*rpa-1*	UUUCAGAUAGUGAAAGAUGGGUUUUAGAGCUAUGCU
ssODNs	
*3XFLAG::rpa-2*	TCTCGCATTTCTAATCTATTTTCATCTTTCCAGAAACTACTAACCATGGAC
	TACAAAGACCATGACGGTGATTATAAAGATCATGATATCGATTACAAGG
	ATGACGATGACAAGTGGAACGAGACTGTCGAGCACGAGAACGCAGGAAA
*3XFLAG::rpa-4*	CTGCCATTTTGTATCATTTCAGCGCGACAGAAAACACGAACAATGGACTA
	CAAAGACCATGACGGTGATTATAAAGATCATGATATCGATTACAAGGATG
	ACGATGACAAGGAGTTCGATCGAAATTTTGACACAAGTGATGGATGGTCA
	TCGCATGATATTTCACAAGAGAGAAAGGTACA
	CGGATGGGCC
*MYC::rpa-4*	CTGCCATTTTGTATCATTTCAGCGCGACAGAAAACACGAACAATGGAACA
	AAAACTGATATCAGAAGAGGATCTGGAGTTCGATCGAAATTTTGACACAA
	GTGATGGATGGTCATCGCATGATATTTCACAAGAGAGAAAGGTACA
*OLLAS::RPA-1*	TTCCCCAATTTTTATGTATCTGTTTCAGATAGTGAAAGATGTCCGGATTCGCCAACGAGCTCGGACCACGTCTCATGGGAAAGGCGGCAATTCACATCAATCACGATGTCTTCAATAA

All tagged lines showed no observable mutant phenotype on the wild-type background. However, OLLAS::RPA-1 showed a small increase in the length of pachytene region past the bend region when introduced to the *rpa-2* mutant background. No effect was observed on the number of diakinesis oocytes.

### Western Blot

Protein samples had SDS urea lysis buffer and 2-mercaptoethanol added, and were immediately boiled for 5 min. After boiling, samples were vortexed for 2 min before being transferred to ice. Samples were run on a 10% SDS Express plus PAGE gel (#M01012; GenScript) using SDS-MOPS buffer. Proteins were transferred to a nitrocellulose membrane in tris–gylcine buffer, washed briefly in 1× PBS–tween, and blocked with 5% dehydrated milk in 1× PBS for 1–2 h. 1× PBST was used as wash buffer. Primary antibodies were diluted in 1× PBS–tween + milk overnight at 4°C. The following morning, the membrane was washed three times using 1× PBS–tween before 2 h incubation with secondary antibody, followed by three more washes. Using WesternBright ECL (#K-12045-D20; Advansta), blots were exposed using the LI-COR Odyssey Infrared Imaging System. Antibodies used were as follows:

Rat α-OLLAS (NOVUS, NBP1-06713, 1:2000), Peroxidase AffiniPure Goat α-Rat IgG (H+L) (1:5000)
Mouse α-FLAG 1:2000 (Sigma F1803), Goat α-Mouse IgG (Kappa light chain) HRP (1:1000)

### Antibody staining and image acquisition

10–20 worms were dissected using a #10 razor blade in M9 on a coverslip. Immediately after gonads were extruded, coverslip was transferred to a positively charged slide and flash frozen on dry ice. Preparation of *ced-1::gfp* worms was performed such that slides were kept in the dark for as long as possible. The coverslips were removed and slides were dipped in methanol for 1 min, followed by a 30 min fix (25 min for *ced-1::gfp* worms) in 4% paraformaldehyde (Alpha Aeser) made from 37% stock. Slides were dipped in 1× PBS–Tween (1× PBST) for 10 min, and then incubated with 0.5%BSA in 1× PBST for 1–2 h. After BSA treatment, slides were incubated with the primary antibody overnight at room temperature. The following day, slides were washed in 1× PBST three times, incubated with secondary antibody for 2 h at room temperature in the dark, and then washed in 1× PBST. Slides were then incubated in the dark with a 4′,6-diamidino-2-phenylindole (DAPI, 1:10 000 of 5 mg/ml stock in 1× PBST), followed by a final wash in 1× PBST. Slides were sealed with VECTASHIELD (Vector Laboratories) and stored at 4°C. The following concentrations of antibodies were used:

Mouse α-FLAG (Sigma F1803, 1:500), Alexa Fluor 488 α-mouse (Invitrogen A21202, 1:500)
Mouse α-FLAG (Sigma F1803, 1:500), DyLight 550 α-mouse (Invitrogen SA5-10167, 1:500)
Rabbit α-RAD-51 (Custom made by Genscript for the Smolikove lab, 1:30 000), Alexa Fluor 488 α-mouse (Invitrogen A21202, 1:500) (Invitrogen A31570, 1:500)
Rat α-OLLAS (NOVUS NBP1-06713, 1:75), Alexa Fluor 594 α-rat (Jackson ImmunoResearch AB_2340689, 1:2000)
Rabbit α-OLLAS(Genscript A01658,1:1000), Alexa Fluor 488 α-rabbit (Molecular Probes/Invitrogene, A32790, 1:500)
Rabbit α-PCN-1 (Gift from M. Michael,1:13000), Alexa Fluor 488 α-rabbit (Molecular Probes/Invitrogene, A32790, 1:500)
Rabbit α-SUN-1 (Novus 41970002, 1:1000), Alexa Fluor 488 α-rabbit (Molecular Probes/Invitrogene, A32790 1:500)
rabbit α-pH3 (Upstate Biotechnologies 06-570, 1:5000), Alexa Fluor 488 α-rabbit (Molecular Probes/Invitrogene, A32790, 1:500)
Mouse α-MAPK-YT (Sigma M9692, 1:400), Alexa Fluor 488 α-mouse (Invitrogen A21202, 1:500)
Rabbit α-MYC (Cell signaling #2278, 1:300), Alexa Fluor 488 α-rabbit (Molecular Probes/Invitrogene, A32790, 1:500)
Mouse α-FLAG (Sigma F1803, 1:500), Alexa Fluor Plus 647 α-mouse (Invitrogen A32787, 1:500)

All images were taken using the DeltaVision wide-field fluorescence microscope (GE lifesciences) with 100×/1.4 NA oil Olympus objective. Images were deconvolved with softWoRx software (Applied Precision) unless otherwise noted. Example images are shown in Supplementary Figures S1–S3.

### Carnoy's and ethanol fix

For whole-worm mutant comparisons and CED-1::GFP engulfment analysis, worms of the indicated ages were placed on an uncharged slide (Surgipath Leica) inside a drop of M9. The M9 was removed using Whatman filter paper, and 8 μl of 95% ethanol (Millipore) for CED-1::GFP analysis, or Carnoy's fix (60% ethanol, 30% chloroform, 10% glacial acetic acid) for gonad length comparison was added to the worms, and then allowed to evaporate. To preserve the worms and stain chromatin, 9 μl of Vectashield with DAPI was added to the slide, and a #1.5 coverslip was placed on top before sealing with acrylic nail hardener.

### Whole worm imaging

Images were taken on a Leica DMRBE microscope using a 10×/0.30 PL FLUOTAR objective. A QIClick (QIMAGING) camera captured images using Q-Capture software. Scale bars in the whole worm images represent 50 μm.

### Nuclear volume

16-bit non-deconvolved images of SUN-1 antibody-stained gonads were taken with the DeltaVision microscope at 100× magnification in 0.1 μm slices. The area at the largest part of each nucleus (a), and the number of slices (b) from top to bottom of each nucleus was measured for five nuclei, and from at least three gonads for each genotype. Using the following formula, volume was estimated based on an ellipsoid volume calculation:

$$\begin{equation*}V = 4/3\pi (a/\pi )(b \times 0.05)\end{equation*}$$

### Immunoprecipitation

Worms were chunked to 10 OP50 seeded NGM plates and allowed to grow for 3–4 days before being washed off with M9 into a 15 ml conical tube. Worms were pelleted and washed with M9 until bacteria was removed (∼5 times). Lysis was performed in a Precelly^®^ machine with equal volumes of Pierce^®^ IP Lysis buffer (Thermo, #8778). The resulting slurry was pelleted, and the supernatant was added to a tube containing 50 μl of Anti-FLAG^®^ M2 Magnetic Beads (Sigma, #m8823). This mix was allowed to incubate on a rotating mixer at 4°C overnight. After overnight incubation, the beads were pelleted using a magnetic stand, and the supernatant was collected. The beads were washed five times using PBS+ ROCHE cOmplete™ Protease Inhibitor Cocktail tablet. Bound proteins were eluted with Thermo Gentle Ag/Ab elution buffer (#21027) and Thermo IgG Elution Buffer (#21004) for 5 min each.

### Quantification of engulfment using CED-1::GFP

Images of *ced-1::gfp* were analyzed using softWoRx software (Applied Precision), and engulfed nuclei were scored only if there was complete engulfment.

### HU treatment

One-day-old worms were transferred to OP50 seeded and sterilized 40 mM HU plates that were made the same day. Worms were allowed to incubate at 20°C for 20 h before transfer to OP50 seeded plates. To determine viability, worms were counted for 3 days as described above.

### Gamma Irradiation (IR)

L4s were transferred to OP50 seeded NGM plates, then exposed to a cesium source 24 h after transfer. For generating DSBs and analysis of RPA-4 focus formation, 120 gray was used, and for viability of *rpa-4* mutants, 100 gray was used. For RPA-4 localization, gonads were dissected 6 h after exposure to IR. For gonad length and apoptosis analysis, worms were allowed to recover for 24 h after treatment.

### Focus quantification

Protocols for focus quantification were based on RAD-51 foci quantification protocols (47,48). Foci were quantified in SoftWoRx software using deconvolved images in all experiments except ones using PCN-1. Background staining was eliminated by reducing channel intensity until staining was only visible within nuclei. Often this reduced the numbers of foci reported to levels below what we expect from looking at un-corrected images. Only foci that were colocalized with DAPI are reported. When quantifying RAD-51 focus abundance, up to five background foci were allowed before quantification. For PCN-1 staining and PCN-1/RPA-2 co-staining, non-deconvolved images were used. When performing staining using rabbit α-OLLAS staining background staining was observed in the distal tip cell, as well as seam cells. Therefore, background was corrected for staining within the gonad (cytoplasmic). Colocalization was identified when a focus was observed on both antibody channels. An example for focus intensity before and after normalization is shown in Supplementary Figure S3C. Normalization removes foci with intensity close to that of background foci (anti-FLAG or anti-OLLAS staining in wild-type, untagged strains). All RPA and RAD-51 figures presented here are adjusted this way.

### Length measurements

Using FIJI, length intensity measurements were gathered by drawing a line through the middle of the gonad from either the bend of the gonad to the first diakinesis nucleus, or from the distal tip to the last diakinesis nucleus, depending on the analysis.

### Microirradiation (MIR)

MIR was performed and analyzed according to the protocol outlined in (49,50) with six nuclei targeted per germline, 3–6 intact germlines scored per condition, with the following alterations: (i) two worms were placed on each live imaging slide, (ii) for RPA-1 localization imaging was performed at 2-min intervals for 1 h, and (iii) for RAD-51 localization imaging was performed at 2-min intervals for 45 min with 10% light source intensity, and 250 ms exposure in the GFP channel. All experiments were performed at 15% pulse intensity. For *gfp11::rpa-1; gfp1–10*, worms were grown at 25°C, L4s were selected from these plates, and then allowed to grow at 20°C until used in the experiment the next day as 1-day-old adults.

### Antibody labeling for single-molecule pull down (SiMPull) analysis

The following antibodies and reagents were used: mouse α-FLAG (Sigma F1803); rat α-OLLAS (Novus Nbp1–06713); rabbit α-MYC (Sigma C3956); EZ-Link NHS-biotin (ThermoFisher Scientific 20217); Cy3 mono-reactive NHS ester (Millipore Sigma PA13105); Cy5 mono-reactive NHS ester (Millipore Sigma PA15101); an Amersham protocol for conjugating NHS reactive reagents was used but modified with the following steps. Zeba desalting columns (0.5 ml) were spun at 1500g to remove storage buffer. The columns were equilibrated with 1 M NaHCO_3_, after which 100 μl of antibody solution was added. The antibodies were spun at 1500g for 2 min. The solution was recovered, and protein concentration was measured using Nanodrop-2000. The equation to calculate the protein concentration at 280 nm was *A* = ϵc*L*, where *A* is the absorbance at 280 nm, ϵ is the molar extinction coefficient for the antibodies (∼170 000 M^−1^cm^−1^), and *L* is the path length which was 1 cm. The volume of NHS reagent (Cy3, Cy5 or NHS-biotin) was calculated so that there would be 20:1 reagent/antibody ratio in the reaction mix. The reagent and antibody were then combined in a 1.5 ml centrifuge tube, mixed and allowed to incubate at room temperature for 1 h. After incubation another spin column was prepped using the same method as above, however the spin columns were equilibrated with DPBS. The antibody/dye solution was spun down and the flow through was collected. The absorbance spectra were then taken using Nanodrop to determine the protein concentration and respective dye-to-antibody ratio.

### Preparation of *C. elegans* lysate for single-molecule studies

Worms were grown on OP50 lawns and chunked 4 days before being rinsed from plates using M9. Pierce™ IP Lysis Buffer (Thermo) was prepared with one tab of protease inhibitor for 10 ml of buffer. After rinsing worms from plates using M9, worms were pelleted by centrifugation at 1600g for 20 s, and supernatant was removed. Lysis buffer was added 1:1 to worm pellet. One milliliter of this mix was placed into Precellys lysis tubes (VK05). Lysis was performed using a Precellys machine for 15 s, before putting the sample on ice. This procedure was repeated three more times, and samples were spun at 2000g for 5 min at 4°C. Supernatant was collected and stored at 4°C.

### SiMPull

Reagent buffer (RB) was used for diluting non-fluorescently labeled antibodies or cell lysate. The RB consisted of [1 mg/ml BSA, 0.01% v/v Triton X-100 and DPBS (Gibco)]. Imaging buffer (IB) was used to dilute fluorescently labelled antibodies and for washing flow cells when fluorescent reagents were present. IB consisted of [25% RB v/v, Gloxy (0.04 mg/ml), 0.8% glucose and Trolox]. Twelve mM trolox was prepared by dissolving Trolox (6-hydroxy-2,5,7,8-tetramethylchromane-2-carboxylic acid, Sigma-Aldrich, MO, USA; 238813-1G) in 12 mM NaOH. The solution was then rotated under a fluorescent light (Sylvania FM13W/835) for 3 days or until the absorbance at 400 nm was ∼0.119. Gloxy was made by mixing (40 mg/ml) catalase in T50. Then 10 μl of this solution was mixed with 90 μl (10 mg/ml) glucose oxidase. Buffers were stored at 4°C and were allowed to reach room temperature prior to using in experiments.

SiMPull experiments (51) were carried out using homebuilt total internal reflection fluorescence microscopy system (TIRFM). The TIRFM system and the flow cells’ preparation are described in (52). A diode-pumped solid state (DPSS) green (532 nm) and red (645 nm) lasers (Coherent, CA, USA) were used to excite the Cy3 and Cy5 dyes, respectively. The laser power output was set to 45 mW for all images. A dual band pass filter (Semrock, NY, USA; FF01-577/690) was used to filter scattered light in the optical path. The fluorescence was collected using a Chroma ET605/70m filter. Movies were taken using an electron-multiplying charge-coupled device (EMCCD) camera (Andor, MA, USA; DU-897-E-CSO-#BV). Exposure setting was 100 ms for all movies. During recording, background was set to 400, and correction to 1200. The gain was set to 295 for all movies.

*C. elegans* lysates from the wild type animals and animals expressing tagged versions of RPA genes were stored in 4°C or kept on ice during experiment until used in the flow cell. After flow cell assembly and TIR acquisition, the flow cell chamber was washed with RB. Neutravidin (0.5 mg/ml solution in RB) was flowed through the chamber and allowed to incubate for 3–5 min. After incubation, the chamber was washed again with RB. Biotinylated antibody (1 nM solution in RB) was then flowed into the chamber. The biotinylated antibody was allowed to incubate in the chamber for 10 min. After incubation the excess antibody was removed by flowing RB through the chamber. *C. elegans* lysate from stock was flowed through the chamber and was allowed incubate for 30 min. Next Cy3- and Cy5-labeled antibodies were mixed in IB + 0.8% glucose to a concentration of ∼10^−11^ M. IB labelled-antibody solution was then flowed through the chamber, the lasers were turned off to ensure that during incubation there was minimal photobleaching of the fluorophores. The labeled antibodies were allowed to incubate with the proteins pulled down from the lysate for 30 min. The chamber was then washed twice with 200 μl of IB + 0.8% glucose. Fluorescence signals originated from the Cy3 and Cy5 dyes were collected by a water immersion 60× objective (Olympus). Several videos were then taken in repetition in unique fields of view (FOV).

Data were quantified using an ImageJ-FIJI plugin (TrackMate). The settings that were used to extract the values were as follows. The difference of Gaussian (DoG) segmenter was used to identify particles. Approximate particle diameter was set to 1.067 μm, and threshold was set to 2. No initial thresholding was used. No filters were set for spots. The Linear Assignment Problem (LAP) Tracker was used to track particles across each image. The maximal distance for frame to frame linking was set to 0.26675 μm and a maximum track segment gap closing value of 0.26675 μm and 2 frames was used. The results were exported, and the data were visualized using Prism 8.0.

### Acridine orange staining

Acridine orange staining was performed on 1-day-old adults. Worms were transferred to a tube of 10 mg/ml acridine orange diluted 1:400 in M9, immediately covered with foil, and allowed to rotate on a mixer for 2 h. After incubation, these worms were transferred to a clean NGM plate. Worms were then placed onto a 10% agarose pad with 8 μl of M9 and 2 μl of Polybead 0.1-μm polystyrene beads (#00876; Polysciences), before imaging on the DeltaVision wide-field fluorescence microscope at 60× magnification (GE lifesciences). Levels of acridine orange-stained nuclei were quantified using softWoRx software (Applied Precision), where nuclei that stain positive were counted towards the total. Example images are shown in Supplementary Figure S3D.

### Statistical analysis

All data were analyzed with Graphpad Prism 8 software. When multiple groups were compared, and non-parametric data used, Kruskal–Wallis tests were applied with multiple comparisons using the two-stage linear step-up procedure of Benjamini, Krieger and Yekutieli to control for false positives. When *q*-values were significant (*q* < 0.05), statistical significance was reported from Mann–Whitney analysis. For the comparison between the nuclear volumes in the PMT, data were normally distributed (according to Anderson–Darling, D’Agostino & Pearson, Shapiro–Wilk and Kolmogorov–Smirnov tests), but variance was not equivalent, therefore a *t*-test with Welch's correction was applied as reported. For complete gonad length measurement, data were normally distributed with equivalent variances, therefore *t*-test was applied and values reported. For SiMPull data multiple *t*-test was used. Since nuclei of the proliferative zone contain replicative nuclei that assemble RPA-1 and RPA-2 in numerous foci that are impossible to count (foci forming a haze coating the DNA), Mann–Whitney could only be performed comparing nuclei with individual foci. For comparing % of nuclei with >20 foci we performed Fisher's exact test. Only statistically significant values are indicated in the figures.

### Sample size

**Figure 1.**
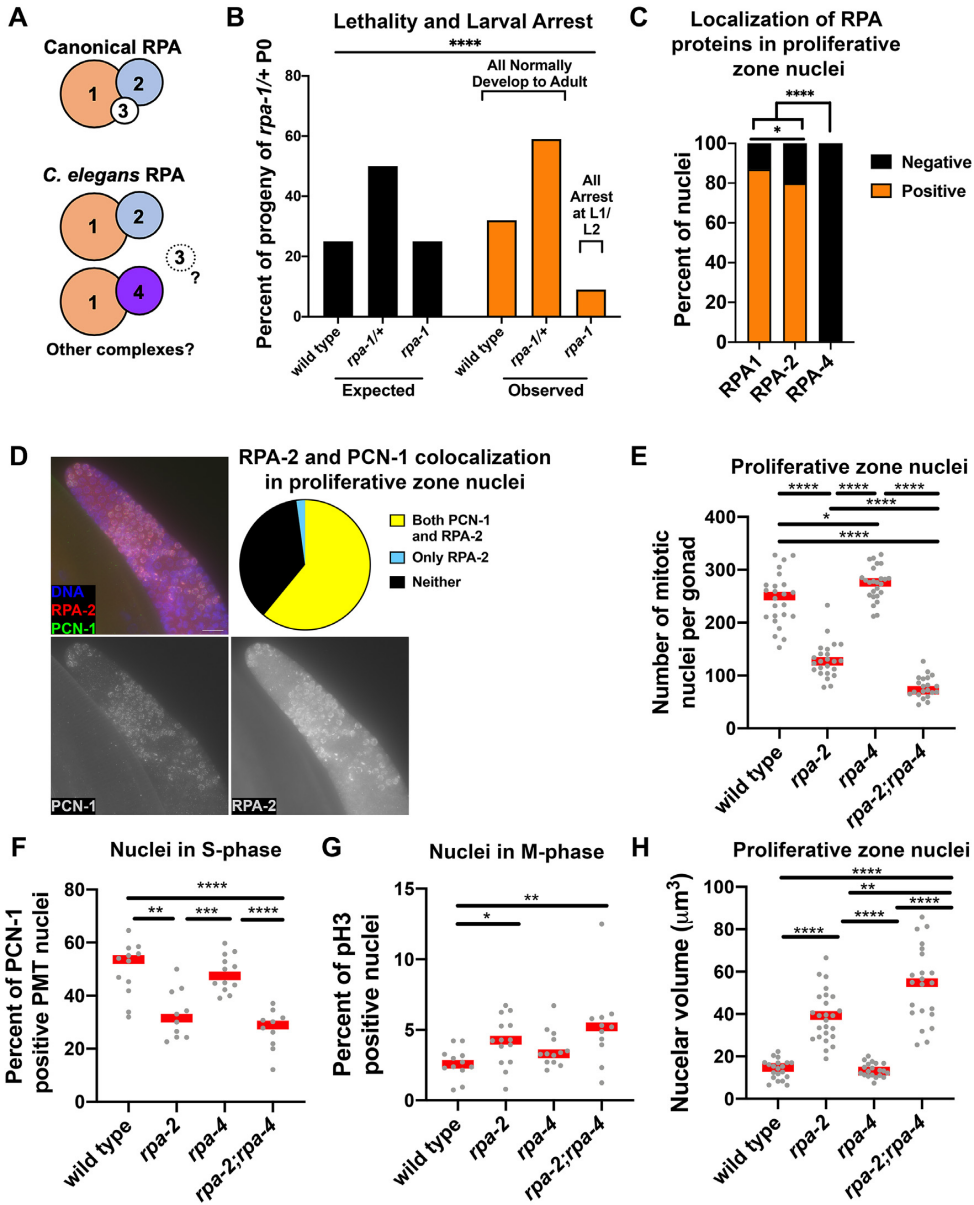
RPA-1 and RPA-2 are involved in replication in the pre-meiotic tip, where mutants of *rpa-2* and not *rpa-4* results in replication defects. (**A**) Model for possible RPA complex combinations in *C. elegans*. RPA-4 and RPA-2 are interchangeable components of the complex, and RPA-3 is unknown. (**B**) Larval lethality and arrest in *rpa-1* mutants (chi-square test performed, *P*-value < 0.0001). (**C**) Proportion of nuclei in the PMT with immunofluorescence staining for each of the tagged RPA subunits. (**D**) Co-staining of gonads with FLAG::RPA-2 and PCN-1 (PCNA ortholog). Scale bar is 10 μm. (**E**) Number of proliferative zone nuclei as counted by position and morphology. (**F**) Percent of nuclei with PCN-1 staining representing S-phase nuclei. (**G**) percent of pH3 (histone H3 phosphorylated at serine 10) positive nuclei representing mitotic index. (**H**) Proliferative zone nuclear volumes as estimated by calculations using FIJI acquired data of SUN-1-stained nuclei (see Materials and Methods). Mann–Whitney tests performed for C, E, F and G, and *t*-test performed with welches correction for H, where *P*-values are represented as ****P < 0.0001, ***P < 0.001, **P < 0.01 and *P < 0.05. Red lines in E–G indicate the median and in H the mean with standard deviation.

Figure 1, panel B assessed viability for n P0s *n* = 7, panel C analysis of *n* gonads/nuclei for OLLAS::RPA-1 *n* = 3/436, FLAG::RPA-2 *n* = 3/219 and FLAG::RPA-4 *n* = 3/198, panel D analysis of *n* gonads/nuclei *n* = 3/233, panel E *n* gonads/nuclei analyzed for wild type *n* = 24/5864, *rpa-2n* = 24/3104, *rpa-4n* = 25/6844 and *rpa-2; rpa-4n* = 21/1636, Panel F analysis of n gonads for wild type *n* = 12, *rpa-2n* = 10, *rpa-4n* = 12 and *rpa-2; rpa-4n* = 10, panel G analysis of *n* gonads for wild type *n* = 12, *rpa-2n* = 14, *rpa-4n* = 13 and *rpa-2; rpa-4n* = 11, panel H analysis of n gonads for wild type *n* = 20, *rpa-2n* = 25, *rpa-4n* = 20 and *rpa-2; rpa-4n* = 22.

**Figure 2.**
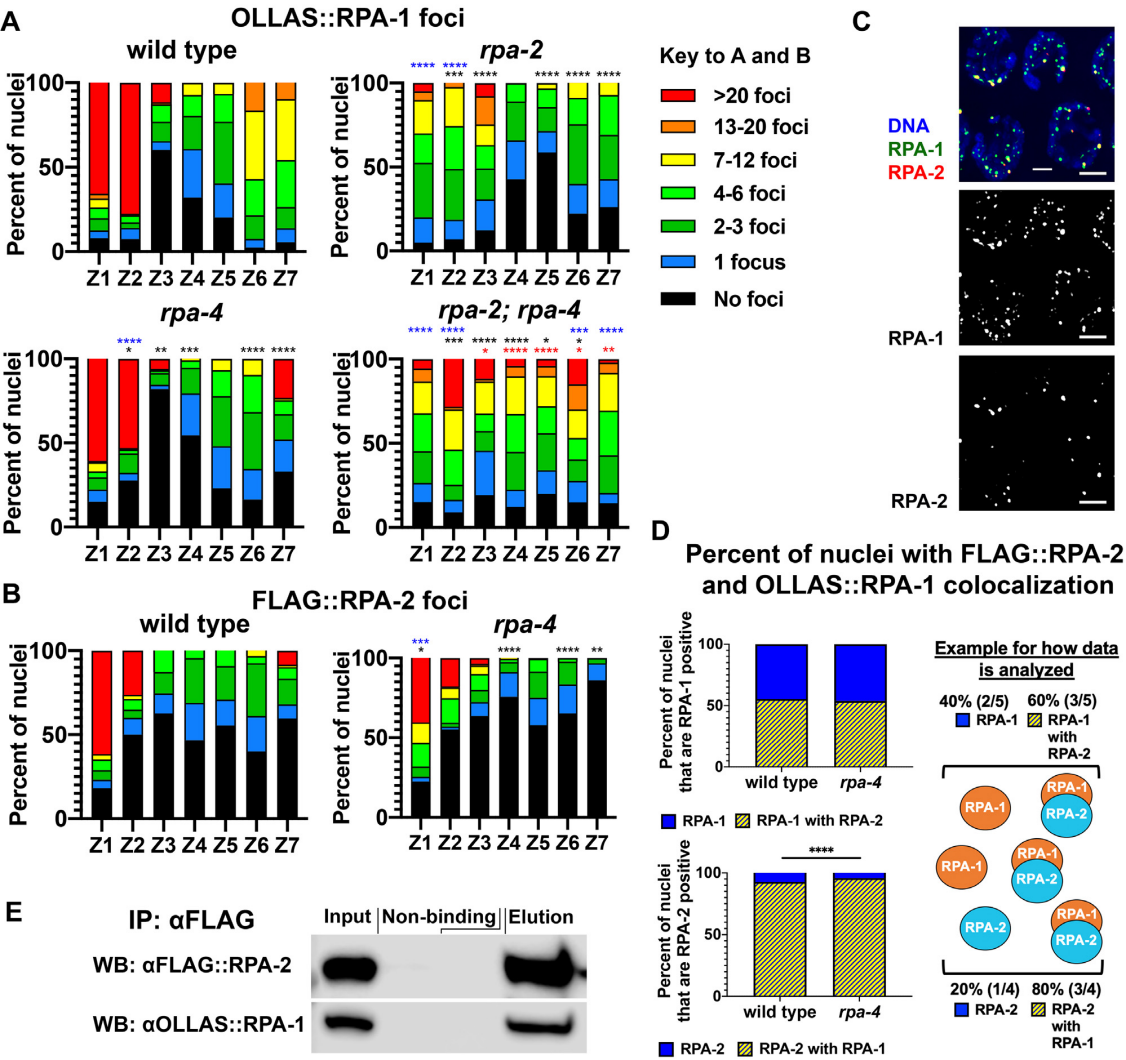
RPA-1 and RPA-2 colocalize and interact *in vitro*; interaction is at DSBs in pachytene. (**A**) Percent of nuclei with indicated amount of OLLAS::RPA-1 foci. Black asterisks are comparison with wild type, and red asterisks are comparison with *rpa-2* mutants, comparing individual foci (<20 foci). Blue asterisks indicate a comparison of >20 foci nuclei between mutants and wild type (for more comparisons, see Supplementary Figure S5D). (**B**) Percent of nuclei with indicated amount of FLAG::RPA-2 foci. Black asterisks represent comparison with wild type, for foci numbers in categories of <20. Blue asterisk represent comparison of >20 versus <20 category (**C**) Image of mid-pachytene nuclei showing colocalization of FLAG::RPA-2 and OLLAS:RPA-1 in otherwise wild-type background. Scale bar is 2 μm. (**D**) Percent of colocalization of FLAG::RPA-2 and OLLAS:RPA-1 in pachytene zones 4–6. A cartoon explaining how the data were quantified is presented on the right. (**E**) Pull down of FLAG::RPA-2 with Co-IP of OLLAS::RPA-1. Mann–Whitney tests performed, where *P*-values are represented as ****P < 0.0001, ***P < 0.001, **P < 0.01 and *P < 0.05.

Figure 2, panel A analysis of *n* gonads/nuclei for wild type *n* = 3/389, *rpa-2n* = 3/291, *rpa-4n* = 3/479, *rpa-2; rpa-4n* = 4/294, panel B analysis of *n* gonads/nuclei for wild type *n* = 3/735, rpa-4 *n* = 3/998, analysis of n gonads/nuclei for wild type *n* = 3/98, *rpa-4n* = 3/193.

**Figure 3.**
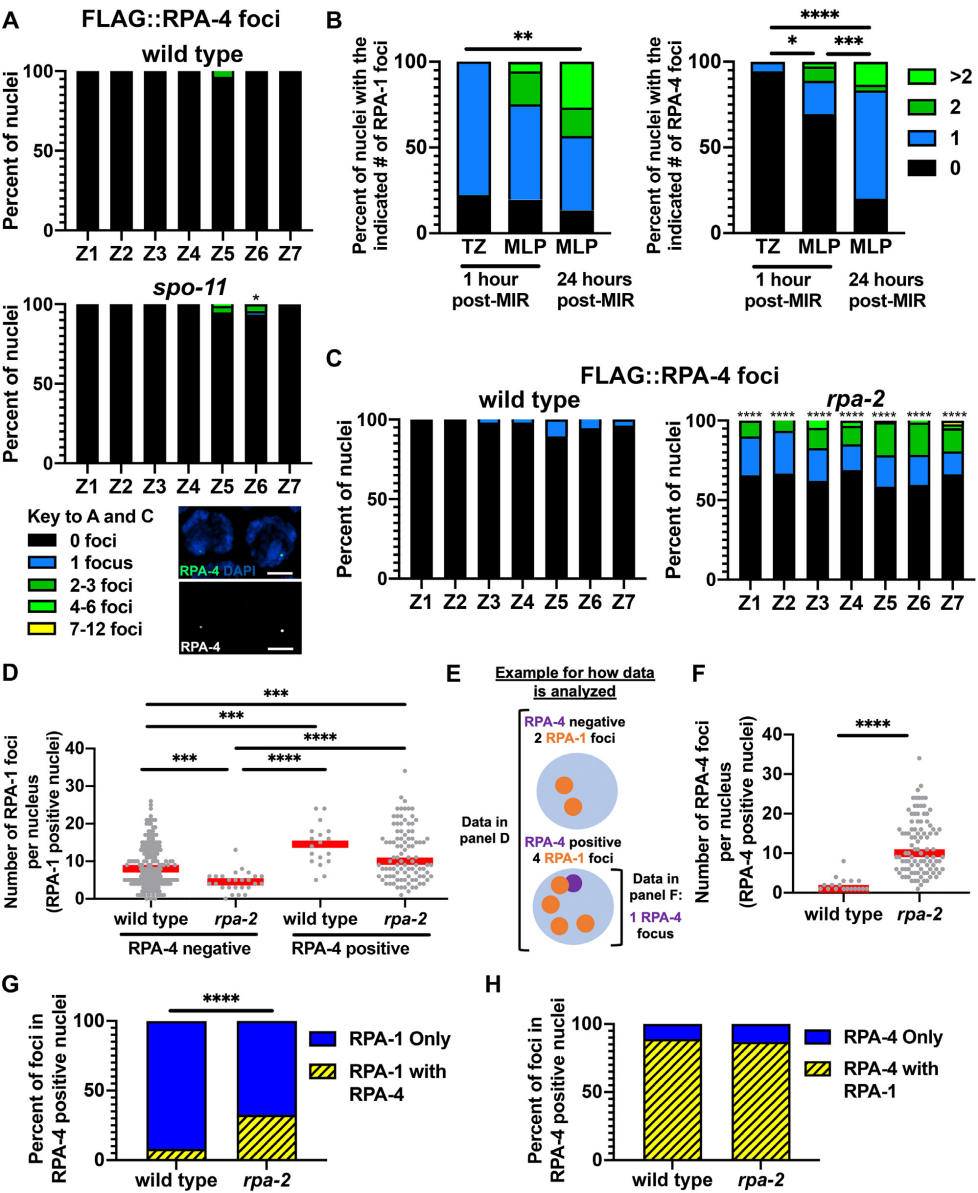
RPA-4 forms rare foci in wild-type worms that become more abundant in *rpa-2* mutants, and localize to SPO-11-independent and exogenous DSBs following RPA-1. (**A**) Percent of FLAG::RPA-4 foci in wild type and *spo-11* mutants. An adjusted image of RPA-4-positive nuclei is presented below. Scale bar is 2 μm. (**B**) Abundance of OLLAS::RPA-1 and FLAG::RPA-4 MIR foci in indicated zones and time periods in *ollas::rpa-1; flag::rpa-4* gonads, out of all MIR nuclei targeted. Indicated zone on x-axis refers to location of nuclei analyzed. For TZ 1 h post-MIR and MLP 24 h post-MIR, TZ nuclei were targeted for laser induced DSBs, while for MLP 1 h post-MIR, MLP nuclei were targeted for laser induced DSBs. (**C**) Percent of nuclei with indicated number of FLAG::RPA-4 foci in wild type and *rpa-2* mutant gonads. (**D**) number of RPA-1 foci in RPA-4-positive and negative mid-pachytene nuclei. Red lines indicate the median. (**E**) A cartoon representing how the data in D and F were quantified. (**F**) number of RPA-4 foci in RPA-4-positive and negative mid-pachytene nuclei. Red lines indicate the median. (**G** and **H**) Percent of FLAG::RPA-4 and OLLAS::RPA-1 foci in mid-pachytene nuclei that colocalize in RPA-4-positive nuclei. Please refer to the cartoon in 2D for how the data were quantified (replace RPA-2 with RPA-4). Mann–Whitney tests performed, where *P*-values are represented as ****P < 0.0001, ***P < 0.001, **P < 0.01 and *P < 0.05.

Figure 3, panel A analysis of *n* gonads/nuclei for wild type *n* = 3/649, spo-11 *n* = 4/940, panel B analysis of *n* gonads/nuclei for 1 h post-IR in TZ *n* = 3/18, 1 h post-IR in MP *n* = 6/36, 24 h post-IR in TZ *n* = 5/30, panel analysis of n gonads/nuclei for wild type *n* = 3/717 and *rpa-2n* = 3/610, panel D A analysis of *n* gonads for wild type *n* = 12 and *rpa-2n* = 11, with *n* = 192 RPA-4-negative wild type nuclei and *n* = 26 *rpa-2* nuclei and RPA-4-positive wild type nuclei *n* = 18 and *rpa-2n* = 97, panel F with number of gonads/nuclei in wild type *n* = 12/18 and *rpa-2n* = 11/98, panel G analysis of n foci for wild type *n* = 101 and for *rpa-2n* = 828, panel H analysis of *n* foci for wild type *n* = 9 for wild type and *rpa-2n* = 306.

**Figure 4.**
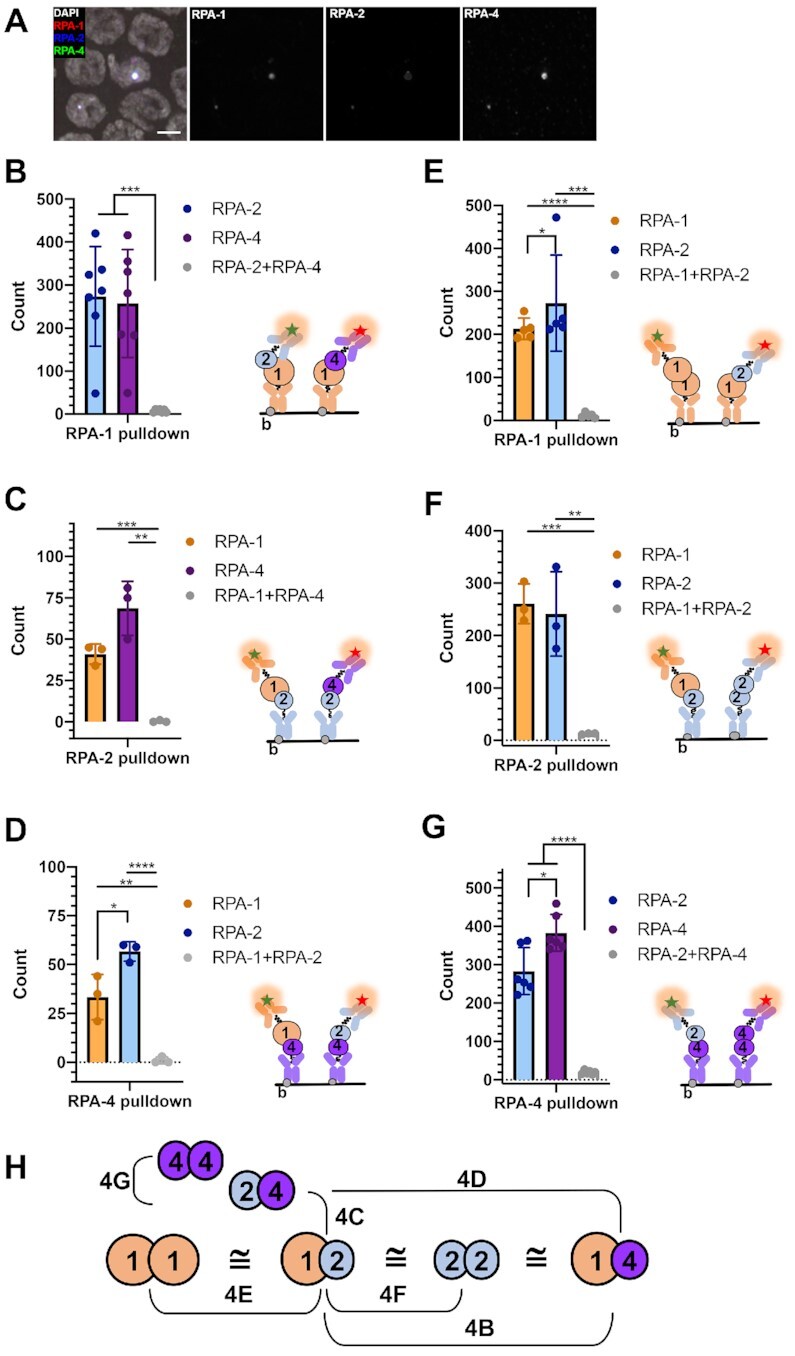
Single-molecule pulldown reveals presence of three possible complex arrangements. (**A**) Colocalization in mid-pachytene nuclei of OLLAS::RPA-1, FLAG::RPA-2 and MYC::RPA-4, in 3-day-old germline of wild type worms. (**B**) Number of RPA-2 and RPA-4 pull down counts through interaction with surface-tethered RPA-1. (**C**) Number of RPA-1 and RPA-4 pull down counts through interaction with RPA-2. (**D**) Number of RPA-1 and RPA-2 pull down counts through interaction with RPA-4. (**E**) Number of RPA-1 and RPA-2 pull down counts through interaction with RPA-1. (**F**) Number of RPA-1 and RPA-2 pull down counts through interaction with RPA-2. (**G**) Number of RPA-2 and RPA-4 pull down counts through interaction with RPA-4. In each panel, the surface immobilization and imaging strategy is shown as a cartoon. The data are shown for at least three independent experiments. In all panels RPA-1, which is being detected using fluorescently labeled anti-OLLAS antibody is shown in orange, RPA-2 (anti-FLAG antibody) is shown blue and RPA-4 (anti-MYC antibody) is shown in purple. Pre-Ab is a control for non-specific signals in the flow cell treated with respective biotinylated antibody and worm extract. Wild type (WT) control reflects the experiments using worms expressing un-tagged RPA forms. The data for each pulled pair were compared using multiple t tests. The respective *P* value is shown under the graph. (**H**) Model interpretation of the results, as related to the panels indicated. *t*-tests performed, where *P*-values are represented as ****P < 0.0001, ***P < 0.001, **P < 0.01 and *P < 0.05.

Figure 4, panel B shows analysis of *n* = 7 experiments with *n* pulldown counts for RPA-1/RPA-2 *n* = 1916, for RPA-1/RPA-4 *n* = 1810, and for RPA-1/RPA-2/RPA-4 *n* = 51, panel C shows analysis of *n* = 3 experiments with *n* pulldown counts for RPA-1/RPA-2 *n* = 123, for RPA-2/RPA-4 *n* = 206, and for RPA-1/RPA-2/RPA-4 *n* = 1, panel D shows analysis of *n* = 3 experiments with *n* pulldown counts for RPA-1/RPA-4 *n* = 100, for RPA-2/RPA-4 *n* = 170, and for RPA-1/RPA-2/RPA-4 *n* = 1, panel E shows analysis of *n* = 3 experiments with *n* pulldown counts for RPA-1/RPA-1 *n* = 1065, for RPA-1/RPA-3 *n* = 1363 and for RPA-1/RPA-1/RPA-2 *n* = 56, panel F shows analysis of *n* = 3 experiments with *n* pulldown counts for RPA-1/RPA-2 *n* = 782, for RPA-2/RPA-2 *n* = 724, and for RPA-1/RPA-2/RPA-2 *n* = 37, panel G shows analysis of *n* = 6 experiments with *n* pulldown counts for RPA-2/RPA-4 *n* = 1699, for RPA-4/RPA-4 *n* = 2300, and for RPA-2/RPA-4/RPA-4 *n* = 117.

**Figure 5.**
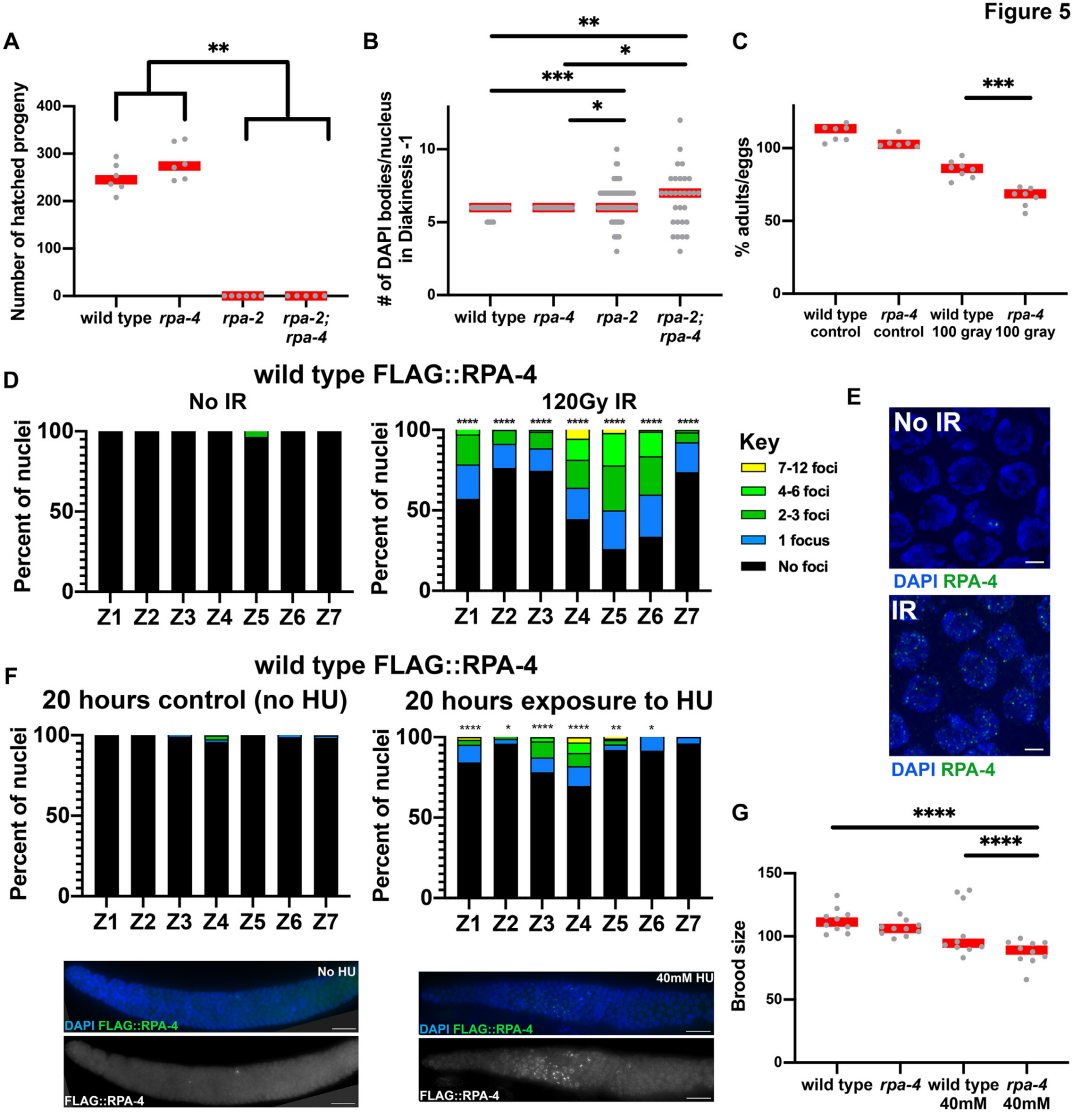
Deletion of *rpa-2* and not *rpa-4* leads to defects in meiotic HR, FLAG::RPA-4 localizes to exogenously induced DSBs, and *rpa-4* deletion leads to HU and IR sensitivity. (**A**) Brood size (number of viable progeny) for each mutant genotype, each point represents the number of adult progeny from a single parent. (**B**) Number of DAPI bodies counted in diakinesis –1 nuclei. (**C**) Percent viability of eggs laid in wild type and *rpa-4* mutants that have been irradiated with 100 Gy gamma-IR as young adult worms and the respective controls. (**D**) Percent of nuclei with the indicated number of FLAG::RPA-4 foci for gamma irradiated young-adult germlines dissected 6 h after irradiation. (**E**) Representative image of mid-pachytene nuclei with and without gamma-IR. Scale bar is 2 μm. (**F**) On the left- representative images of FLAG::RPA-4 gonad dissected and stained 20 h after treatment with 40 mM HU and 3-day-old adult not treated with HU. On the right- FLAG::RPA-4 foci in HU treated germlines, on the left worms grown for the same amount of time without HU. Black stars are comparison between the two graphs. Scale bar is 20 μm (**G**) Brood size of HU treated wild type and *rpa-4* mutant worms. Mann–Whitney tests performed, where *P*-values are represented as ****P < 0.0001, ***P < 0.001, **P < 0.01 and *P < 0.05. Red lines in A–C and G indicate the median.

Figure 5, panel A analysis of n P0s for wild type *n* = 6, *rpa-2n* = 6, *rpa-4n* = 6, *rpa-2; rpa-4n* = 5, panel B analysis of *n* diakinesis – 1 nuclei for wild type *n* = 56, *rpa-2n* = 88, *rpa-4n* = 35, *rpa-2; rpa-4n* = 30, panel C analysis of n P0s for wild type control *n* = 7, *rpa-4* control *n* = 6, wild type 100 gray *n* = 8, *rpa-4* 100 gray *n* = 7, panel D analysis of n gonads/nuclei for no IR *n* = 3/649 and 120 gray *n* = 3/590, panel F number of gonads/nuclei analyzed for no HU treatment *n* = 3/736 and HU treated worms *n* = 3/662, panel G analysis of n P0s for wild type control *n* = 10, *rpa-4* control *n* = 10, wild type HU treated *n* = 10, and *rpa-4* HU treated n = 10.

**Figure 6.**
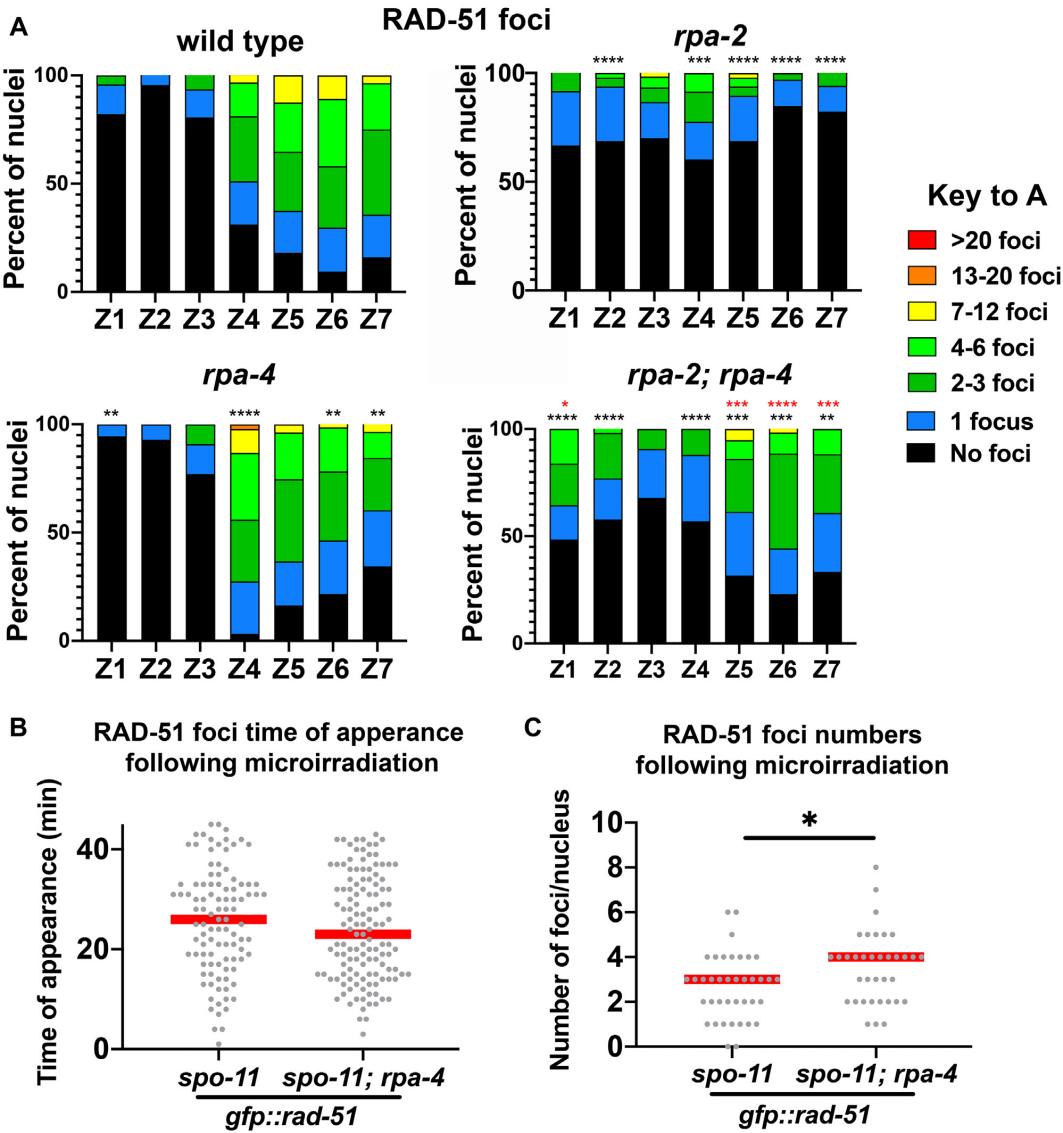
*rpa-2* and *rpa-2; rpa-4* mutants have decreased RAD-51 foci compared to wild type, and RAD-51 MIR foci are more abundant in *rpa-4* mutants. (**A**) Percent of nuclei with indicated amount of RAD-51 foci. Black asterisks are comparison with wild type, and red asterisks are comparison with *rpa-2* mutants. (Mann-Whitney tests performed, where *P*-values are represented as ****P < 0.0001, ***P < 0.001, **P <0.01 and *P < 0.05, where black asterisks represent comparison with wild-type, and red asterisks represent comparison with *rpa-2*). (**B**) Time of appearance of GFP::RAD-51 foci in *spo-11* mutant background after treatment with UV laser MIR. Mann-Whitney, *P*-value = 0.0157. (**C**) Number of GFP::RAD-51 foci following treatment with UV laser MIR. Mann–Whitney, *P*-value = 0.0847. Red lines in B and C indicate the median.

Figure 6, panel A analysis of *n* gonads/nuclei for wild type *n* = 4/610, *rpa-2n* = 4/300, *rpa-4n* = 4/606, *rpa-2;rpa-4n* = 5/375, panel B analysis of *n* foci for *spo-11 =* 101, *spo-11; rpa-4 =* 128, panel C analysis of n nuclei for *spo-11* = 39, *spo-11;rpa-4* = 36.

**Figure 7.**
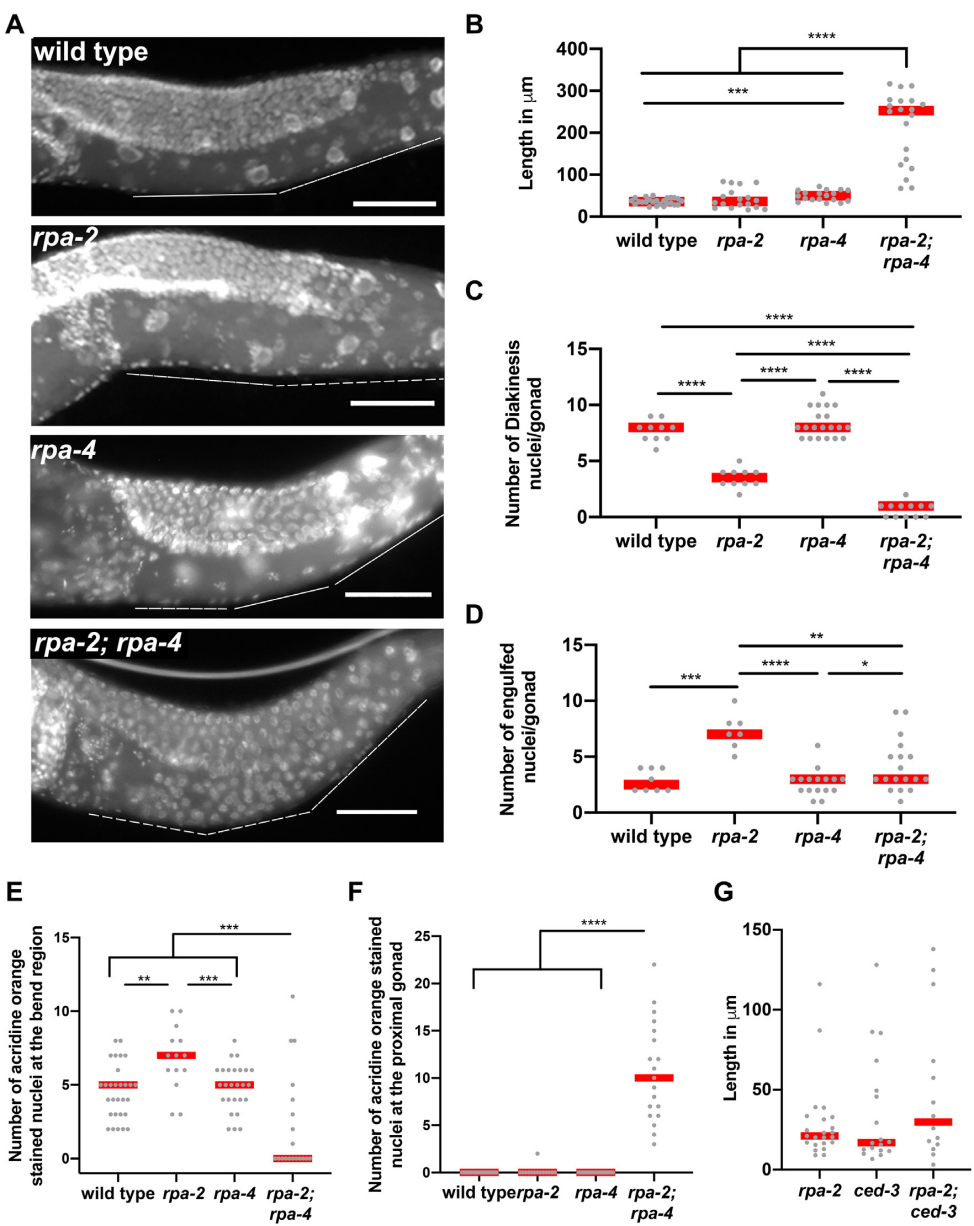
Double deletion of *rpa-2* and *rpa-4* leads to meiotic progression defects, with defects in apoptosis. (**A**) Representative images of gonads in Carnoy's fixed whole worms. Scale bar is 50 μm. (**B**) Length of pachytene extension in indicated mutants measured from bend to first diakinesis nucleus. (**C**) Number of diakinesis oocytes in each mutant background. (**D**) Number of CED-1::GFP engulfed nuclei in indicated mutants of 1-day-old worms. (**E**) Number of acridine orange staining nuclei in distal gonad measured until the end of the ‘bend’ region. (**F**) Number of acridine orange staining nuclei in proximal gonad measured from the end of the ‘bend’ region to the spermatheca. (**G**) Length of pachytene extension in indicated mutants from bend to first diakinesis nucleus in 2-day-old adults. Mann–Whitney tests performed, where *P*-values are represented as ****P < 0.0001, ***P < 0.001, **P < 0.01 and *P < 0.05. Red lines in B–F indicate the median.

Figure 7, panel B analysis of n germlines for wild type *n* = 25, *rpa-2* = 28, *rpa-4n* = 18, *rpa-2; rpa-4n* = 20, panel C analysis of *n* germlines for wild type *n* = 10, *rpa-2n*-10, *rpa-4n* = 20, *rpa2; rpa-4n* = 12, panel D analysis of n germlines for wild type *n* = 8, *rpa-2n* = 7, *rpa-4n* = 16, *rpa-2; rpa-4n* = 18, panels E and F analysis of *n* germlines for wild type *n* = 29, *rpa-2n* = 14, *rpa-4n* = 26, *rpa-2; rpa-4n* = 19. panel G shows analysis of *n* gonads for wild type *n* = 25, *rpa-2n* = 23, *ced-3n* = 18, and *rpa-2;ced-3n* = 14.

**Figure 8.**
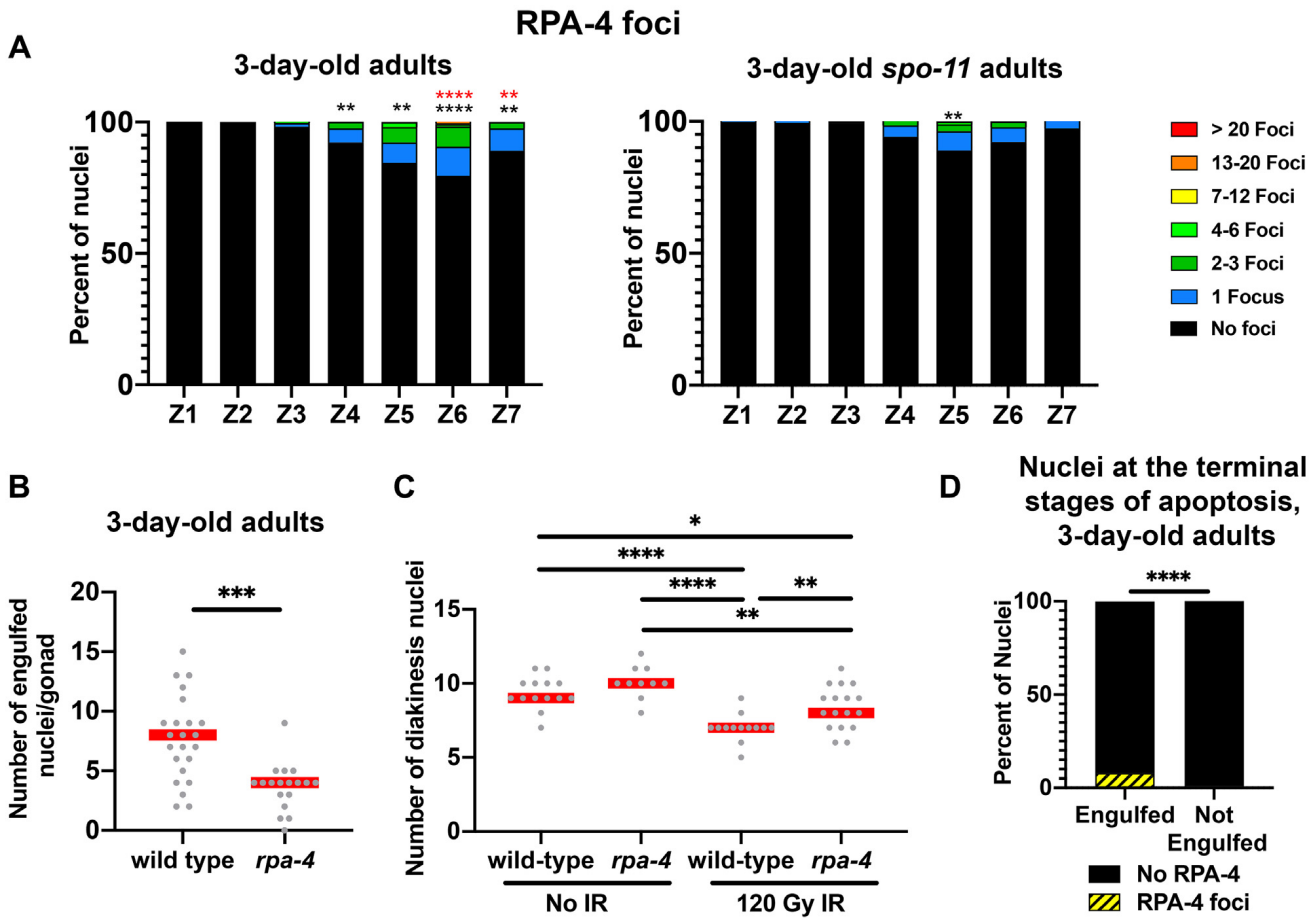
RPA-4 foci appear in greater abundance in aging worms. (**A**) Percent of nuclei with indicated amount of FLAG::RPA-4 foci in 3-day-old hermaphrodites. Black asterisks are comparison with wild type 1-day-old adult worms and red asterisks are comparison with 3-day-old *spo-11* mutants. (**B**) Number of CED-1::GFP engulfed nuclei in wild type and *rpa-4* mutant 3-day-old worms. (**C**) Number of diakinesis oocytes in wild type and *rpa-4* worms in 24 h post-IR and mock-IR. (**D**) Presence of FLAG::RPA-4 or foci in unmarked or CED-1::GFP engulfed nuclei in 3-day-old hermaphrodites. Mann–Whitney tests performed, where *P*-values are represented as ****P < 0.0001, ***P < 0.001, **P < 0.01 and *P < 0.05. Red line in B, C and E indicate the median.

Figure 8, panel A shows analysis of *n* gonads/nuclei for 3-day-old *flag::rpa-4n* = 6/1962, *1-day-old spo-11; flag*::rpa-4 *n* = 940 and 3-day-old *spo-11; flag::rpa-4n* = 6/2089, panel B shows analysis of *n* gonads for *ced-1::gfpn* = 23 and *ced-1::gfp; rpa-4n* = 18, panel C shows analysis of *n* gonads for wild type control *n* = 14, *rpa-4* control *n* = 10, wild type IR treated *n* = 13, and *rpa-4* IR treated *n* = 17, panel D shows analysis of *n* = 18 gonads, *n* = 101 engulfed nuclei and *n* = 828 not engulfed nuclei.

## RESULTS

### RPA-2, but not RPA-4, is required for mitotic replication

The *C. elegans* genome encodes three RPA proteins (RPA-1, RPA-2 and RPA-4) identified by homology to RPA subunits from other organisms (wormbase.org, Figure 1A, Supplementary Figure S4A). RNAi of *rpa-1* results in gonad developmental defects (38) and RNAi of *rpa-2* results in sensitivity to radiation and DNA damage inducing agents (53). The phenotypes observed by RNAi knockdown of genes encoding the RPA subunits may not fully represent the biological requirement for these genes, as this method does not lead to gene knockout. To identify the roles of RPA subunits in *C. elegans* we created CRISPR/Cas9 mediated loss-of-function alleles for *rpa-1* and *rpa-4* and utilized an available deletion mutant for *rpa-2* (Supplementary Figure S4B–D). Consistent with its essential role in replication, an early frameshift mutant of *rpa-1* is larval lethal. Mutants with knockouts of genes essential only for HR in *C. elegans* develop due to maternal contribution to the zygote, but they lay dead eggs. However, none of the *rpa-1* mutants of heterozygote mothers developed past the L2 larval stage, and the number of *rpa-1* L1/L2 larvae were diminished in their numbers (9% versus the expected 25% of progeny, Figure 1B). These observations are expected if RPA-1 is required for replication past the 100-cell stage embryo (when maternal contribution is depleted), and if RPA-1 is an obligatory RPA subunit in replication. In contrast, *rpa-2* and *rpa-4* single mutants as well as *rpa-2; rpa-4* double mutants develop to adults, indicating that these genes are not essential for somatic DNA replication. This result was unexpected, as in other metazoans RPA2 and RPA1 subunits are both essential for replication.

To determine the function of RPA subunits in *C. elegans* meiosis, we N-terminally tagged *rpa-2* and *rpa-4* using CRISPR with 3X FLAG epitopes. RPA-1 was previously N-terminally tagged using the OLLAS epitope, but the localization pattern was not described in detail (35). All epitope-tagged proteins are likely functional, as epitope-tagged strains showed no difference in brood size or egg viability [(35), Supplementary Figure S5A and Materials and Methods]. The RPA complex has a well-established role in replication (16), therefore we examined the localization of the three RPA subunits in nuclei undergoing replication in the germline. The pre-meiotic tip (PMT) contains the stem cell niche of the germline and most nuclei are found in S/G2 phases of the cell cycle (54,55). OLLAS::RPA-1 and FLAG::RPA-2 staining was present in a majority (87% and 80%, respectively) of nuclei of the PMT (Figure 1C), which was similar to the previously reported percent of S-phase nuclei [∼60–70% (54,55)]. Most of these nuclei formed abundant OLLAS::RPA-1 and FLAG::RPA-2 foci co-localizing with chromosomes (DAPI) as a ‘haze’, or ‘coating’ on the DNA and with each other (among ‘haze’ nuclei, 99% of RPA-1 nuclei colocalized with RPA-2 and 100% of RPA-2 nuclei colocalized with RPA-1). To determine if this RPA localization pattern is associated with S-phase nuclei, we co-stained for FLAG::RPA-2 and PCN-1 [Proliferating Cell Nuclear antigen homolog, clamp subunit associated with DNA polymerase during replication, (56,57)]. PCN-1 is found in S-phase nuclei in a similar localization pattern to that of RPA-1 and RPA-2, and all nuclei that expressed PCN-1 staining also stained for FLAG::RPA-2 (Figure 1D). *rpa-4* appears to be a complex duplication of *rpa-2* with several isoforms, the largest product of which is isoform a, whose product shares 30.56% identity to RPA-2. RPA-2 and RPA-4 appear to be orthologs of human RPA-2 based on their protein alignment (58). Therefore, we expected that the two proteins would exhibit a similar localization pattern. However, unlike RPA-2, FLAG::RPA-4 was absent from PMT nuclei (Figure 1C) suggesting that RPA-4 does not play a role in replication.

Next, we examined the role of RPA subunits in germline DNA replication through the analysis of germlines in *rpa-2* and *rpa-4* mutants. Since no worms past the L2 stage were observed in *rpa-1* mutants, we were unable to use this allele to further study RPA-1’s role in the germline, because it is tissue that develops in later larval stages. *rpa-2* and *rpa-4* single mutants as well as *rpa-2*; *rpa-4* double mutants all contained germlines, indicating that neither RPA-2 nor RPA-4 is required for replication leading to germline formation. However, detailed analysis of *rpa-2* mutants uncovered a role for RPA-2 in germline DNA replication. Replicating germline nuclei are found in the proliferative zone located at the distal region of the germline. These nuclei are mitotically/pre-meiotic dividing nuclei and are mostly in S and G2 phases of the cell cycle, with 50–71% of the nuclei in S phase, (55,59,60). We compared the total number of proliferative zone nuclei in the PMT in *rpa-2* knockout worms to that of the wild type worms based on nuclear morphology. In *rpa-2* young-adult hermaphrodites, the total number of proliferative zone nuclei was almost half of the amount that was observed in wild-type germlines (Figure 1E). We tested if this reduction could be attributed to a decrease in the number of S-phase nuclei by quantifying the number of nuclei with PCN-1 staining. The percent of S-phase nuclei in *rpa-2* single mutants and *rpa-2; rpa-4* double mutants was reduced by nearly half, indicating a reduction in nuclei undergoing replication (Figure 1F). Next, we stained and quantified the number of nuclei that were positive for Histone H3 phosphorylated at serine 10 (pH3), which marks mitotic M-phase nuclei (Figure 1G). We observed a small increase in the percent of M-phase nuclei in *rpa-2* mutants, consistent with M-phase arrest triggered by replication errors. Germline nuclei that experience a replication block, such as those that are treated with HU, arrest and increase their nuclear volume (32). In concordance with the presence of replication defects, *rpa-2* mutants had larger nuclei than wild type (Figure 1H and S5B). If replication defects lead to decreased proliferative zone nuclei, it is likely that this could lead to reduction in overall germline size as fewer nuclei enter meiosis. Indeed, gonad length was reduced in *rpa-2* mutants (Supplementary Figure S5C). All together, these results indicate that RPA-2 is essential for normal germline replication.

Since RPA-4 shares homology with RPA-2, it is possible that RPA-4 functions similarly to RPA-2, such that *rpa-4* mutants will phenocopy *rpa-2* mutants. However, RPA-4 localized in few proliferative zone nuclei (Figure 1C), suggesting it may not play a role in germ-cell replication. *rpa-4* mutants were indistinguishable from wild type in the percent of PCN-1-positive nuclei and only showed mild effects on total nuclei numbers or pH3-positive nuclei, supporting our hypothesis that RPA-4 does not play a significant role in replication. In agreement, *rpa-4* mutants did not modify the *rpa-2* phenotypes indicative of an effect on replication, and *rpa-2; rpa-4* double mutants only had small effects on total proliferative zone nuclei number and nuclear volume compared to the *rpa-2* single mutant (PCN-1 or pH3 positive nuclei, Figure 1F and G). *rpa-2; rpa-4* double mutants had longer gonads than *rpa-2* mutants, which we discuss below may be due to a later meiotic role of RPA-4. These data are consistent with limited localization of RPA-4 in proliferative zone nuclei, suggesting that despite its homology to RPA-2, RPA-4 does not play a significant role in replication.

### RPA-1 and RPA-2 colocalize and form a complex that excludes RPA-4 in wild-type pachytene nuclei

During meiotic prophase I, programmed DSBs are formed by SPO-11 and repaired by HR (27), which requires the RPA complex for proper RAD-51 loading (31). RPA-1 was previously shown to localize to germline nuclei; however, the localization of RPA-2 has not been reported. To identify the localization pattern of RPA-2 in meiotic cells undergoing recombination we stained gonads of our 3XFLAG-tagged RPA-2 strain and compared the localization to that of OLLAS-tagged RPA-1. The gonads were divided into seven zones (for details, see Materials and Methods), where zones 1–2 represent mostly the mitotic zones, zone 3 represents mostly the transition zone (leptotene and zygotene), and zones 4–7 represent pachytene. RPA-1 and RPA-2 have similar localization patterns in the *C. elegans* hermaphrodite germline (Figure 2A-C and S2F). In wild-type germlines, RPA-1 and RPA-2 are mostly absent from the transition zone (Z3), where programmed meiotic DSBs form (31). During pachytene, DSBs are resected and RPA binds the ssDNA ends. Interestingly, RPA-1 and RPA-2 foci numbers peaked at mid to late pachytene, which was not expected as RAD-51 (which displaces RPA) focus formation peaked at early to mid-pachytene. However, these results match previous observations of RPA-1 foci in late pachytene in nuclear spreads, which likely mark crossover intermediates (34). We observed in late pachytene/diplotene nuclei a ‘haze’ of RPA-1 and RPA-2 similar to what was observed in the PMT, which we interpret as nuclei preparing for the next round of replication after fertilization. In the pachytene region, RPA-2 foci were less abundant than RPA-1 (1.1 focus/nucleus compared to 4.4 foci/nucleus, respectively), suggesting RPA-1 can bind ssDNA without RPA-2.

In most species, RPA-1 and RPA-2 form a complex required for solubility and stability of RPA-1, and thus RPA-2 is essential for RPA-1 function, including its binding to ssDNA at resected DSBs (61). However, deletion of *rpa-2* reduced, but did not eliminate OLLAS::RPA-1 focus formation (Figure 2A). The most robust effect of *rpa-2* mutants on RPA-1 localization was in mitotic cells (zones 1 and 2), indicating a role for RPA-2 in promoting the interaction of RPA-1 with ssDNA for normal replication, as supported by PCN-1 staining (Figure 1D). *rpa-2* knockout also had an effect on OLLAS::RPA-1 loading in meiotic nuclei, where zones 5–7 have fewer OLLAS::RPA-1 foci than wild type (49%, 68%, and 62% reduction in each respective zone, Figure 2A), indicating that RPA-2 is also required for wild-type levels of RPA-1 localization during DSB repair.

Next, we examined if RPA-1 and RPA-2 co-localize *in vivo* at the time of meiotic DSB repair. FLAG::RPA-2 and OLLAS::RPA-1 foci co-localized extensively, consistent with formation of an RPA complex including these two subunits (Figure 2C and D). Almost all RPA-2 foci colocalized with RPA-1, but not all RPA-1 foci colocalized with RPA-2, in agreement with higher abundance of RPA-1 foci compared to RPA-2 foci. Co-immunoprecipitation (co-IP) supports that this co-localization reflects a physical interaction between RPA-2 and RPA-1, as pull-down of FLAG::RPA-2 resulted in co-IP of OLLAS::RPA-1 (Figure 2E).

While RPA-1 localization to germline nuclei was attenuated in *rpa-4* deletion, this had a small effect compared to the one found in *rpa-2* mutants (Figure 2A). Co-localization of RPA-1 with RPA-2 foci also had minor changes in the *rpa-4* mutant backgrounds compared to wild type (Figure 2D). These data suggest that RPA-4 has a small effect on RPA-1 and its interaction with RPA-2.

### RPA-4 localizes to subset of DSBs, is regulated by RPA-2 and inhibits RPA-1 focus formation

RPA-4 is the ortholog of RPA-2, suggesting it may have a function in HR. Similar to mitotic germline nuclei, RPA-4 was also absent from most meiotic germline nuclei (Figure 3A). Overall, FLAG::RPA-4 was found in <1% of germline nuclei, and almost exclusively in the pachytene stage of meiotic prophase I. The localization of RPA-4 in the form of foci in pachytene nuclei (zones 4–7) suggests that it is recruited to the sites of DNA damage, likely ssDNA. The limited localization of RPA-4 to meiotic nuclei suggests that RPA-4 localized only to a subset of resected DSBs. Germline DSBs are generated by 3 mechanisms: SPO-11-induced DSBs, DNA damage due to replication fork collapse and DNA fragmentation following apoptosis. SPO-11-induced DSBs are the majority of germline DSBs and are found only in early to mid-prophase I. DNA fragmentation following apoptosis is only found in a few nuclei in the late pachytene region of the germline. The localization of RPA-4 to nuclei prior to late pachytene indicates that it is not marking fragmented DNA of apoptotic nuclei (but this does not preclude it from binding DNA of nuclei marked for apoptosis). To test if RPA-4 localization depends on programmed meiotic DSBs, we tested FLAG::RPA-4 localization in *spo-11* mutants. In agreement with its limited localization, RPA-4 foci numbers were not affected by the removal of *spo-11*, indicating that RPA-4 marks SPO-11-independent DSBs (Figure 3A). Therefore, it is likely that under normal growth conditions, RPA-4 marks DSBs created by other forms of DNA damage, possibly replicative damage that is carried over as these nuclei proceed to meiosis.

To test if RPA-4 focus formation is dependent on DNA damage, we performed UV laser MIR in adult hermaphrodite germlines. For this study, we used GFP11-tagged RPA-1 expressed from the endogenous locus to mark DSB sites [previously generated in our lab: (35)]. We found that indeed, GFP11-tagged RPA-1 localized to the site of exogenously-induced DSBs forming ∼1 focus per nucleus and localized to DSBs ∼6 min following MIR (Supplementary Figure S6AB). These findings are consistent with ones observed with a different transgenic line [integrated extra-chromosomal array (49)]. Using an OLLAS-tagged RPA-1, we observed RPA-1 localization that increased 24 hours post-MIR (Figure 3B). RPA-1 MIR foci were detected in higher levels in mid-pachytene nuclei compared to transition zone nuclei (Figure 3B), consistent with previously published data (49). To test if RPA-4 is recruited to exogenously-induced DSBs we analyzed the co-localization of RPA-4 with RPA-1 following laser MIR. Our results indicate that all (100%, n = 54) RPA-4 foci localize to MIR foci containing RPA-1. Unlike RPA-1 which localized to MIR damage in the first hour post-MIR, RPA-4 foci were found in similar numbers only 24 hours following MIR (Figure 3B). Taken together, these data suggest that RPA-4 foci localize to DNA damage, and that RPA-4 foci formation follows RPA-1 localization. RPA-4 localization likely depends on DSB resection, and likely represents an RPA-4 complex with RPA-1.

Given the distinct localization pattern of RPA-2 and its paralog RPA-4, it is possible that the two proteins have different functions. While *rpa-4* had only minor effects on RPA-2 localization, *rpa-2* removal showed a notable effect on RPA-4 localization. In *rpa-2* worms, the number of RPA-4-positive nuclei increased by an average of 16 times compared to wild type (from 8 foci/gonad in wild-type worms to 128 foci/gonad in *rpa-2* worms, Figure 3C), indicating that RPA-2 attenuates RPA-4 focus formation, either directly or indirectly (see Discussion).

To investigate the relationship between RPA-4 and RPA-1, we focused our attention on RPA-4-positive nuclei (Figure 3D–F). RPA-4 foci were only found in nuclei that also contained RPA-1 foci. RPA-4-positive nuclei had slightly higher levels of RPA-1 foci when compared to RPA-4-negative nuclei (14.3 vs 9 foci/nucleus in pachytene) in wild-type worms. However, in *rpa-2* mutants, the effect was bigger [12 versus 4.6 foci/nucleus in pachytene (Figure 3D)]. As evident by the overall distribution of RPA-4 foci (Figure 3A, C), when focusing only on RPA-4-positive nuclei, RPA-4 levels were significantly elevated in the absence of *rpa-2* (12 versus 2 foci/nucleus, Figure 3F). While in RPA-4-positive cells RPA-1 foci were more abundant than RPA-4 foci, almost all RPA-4 foci colocalized with RPA-1 in wild type and *rpa-2* mutants (Figure 3G and H). Altogether these data suggest that RPA-4 focus formation is attenuated by RPA-2, dependent upon significant amounts of unrepaired DNA damage.

### *C. elegans* RPA-1, RPA-2 and RPA-4 form six different complexes

Using co-IP, we have shown that RPA-1 and RPA-2 physically interact (Figure 2E). RPA-4 was not detectable on Western blot analysis due to its low abundance which prevented the analysis of its physical interaction with RPA-2 and RPA-1 by traditional co-IP. All MYC::RPA-4 foci colocalize with OLLAS::RPA-1 and FLAG::RPA-2 (Figure 4A). However, due to the low resolution of immunofluorescence imaging, it is possible that this colocalization indicates formation of mixed ssDNA-RPA filaments composed of separate RPA complexes. To bypass this limitation, we performed Single-Molecule Pull Down [SiMPull (51)] experiments using this triple-tagged strain. The SiMPull experimental strategy involved capture of the RPA complexes from the whole worm lysate by the surface-tethered biotinylated antibodies against a tag on one of the subunits, followed by visualization of the RPA-1, RPA-2 and/or RPA-4 proteins via fluorescent antibodies specific to tags present on each protein. This approach allowed us to enumerate RPA complexes of different compositions present in the same mixture. We expected to see complex formation of RPA-1 with RPA-2 predominately as it was detected in IPs (Figure 2E and Supplementary Figure S6C–E) and because the recombinant RPA-1 and RPA-2, when co-expressed, have been shown to form a one-to-one stoichiometric complex (62). Unexpectedly, we found evidence for formation of three distinct complexes including RPA-1/RPA-2, RPA-1/RPA-4 and RPA-2/RPA-4 (Figure 4B–D) as well as homodimers or homo-oligomers of RPA-1, RPA-2 and RPA-4 (Figure 4E–G, Supplementary Figure S6CD). In very few instances (∼1–4% of observed events), the RPA-1/RPA-2/RPA-4 complexes were also observed. The bar graphs in Figure 4 (B–G) show track counts taken across multiple fields of view for each respective combination. RPA complexes containing OLLAS::RPA-1 were captured from the lysate to the surface-tethered anti-OLLAS antibodies (Figure 4B and E). The presence of 3XFLAG::RPA-2 and MYC::RPA-4 (Figure 4B) or OLLAS::RPA-1 and 3XFLAG::RPA-2 (Figure 4E) was simultaneously detected and quantified using Cy3-labeled anti-FLAG and Cy5-labeled anti-MYC antibodies, or Cy3-labeled ani-OLLAS and Cy5-labeled anti-FLAG antibodies, respectively. Pre-Ab track and wild-type control track counts represent the number of counts before the fluorescent antibodies are added into the microscope flow cell and the control experiment that uses the lysate from the wild-type animals, respectively. Post-Wash values represent the number of tracks measured after the antibody had incubated for 30 min in the presence of lysate and had been washed with imaging buffer, which represents the number of respective RPA complexes. Unexpectedly, we observed RPA-1/RPA-2, RPA-1/RPA-1 and RPA-1/RPA-4 with the same frequency. Then, 3XFLAG::RPA-2 was anchored to the surface and OLLAS::RPA-1 and MYC::RPA-4 were visualized using Cy3 and Cy5-labeled antibodies against OLLAS and MYC, respectively (Figure 4C). Next, we anchored the MYC::RPA-4 and visualized RPA complex formation by adding fluorescent antibodies against OLLAS (RPA-1) and FLAG (RPA-2), which recapitulated our previous results (Figure 4D). In both configurations, we observed RPA-4 preferentially binding RPA-2. The results of the SiMPull experiments were consistent between multiple lysates and no non-specific localization was observed (Supplementary Figure S6C–E). Complexes containing two RPA-2 subunits were as frequent as RPA-1/RPA-2 complexes (Figure 4F) and RPA-4/RPA-4 complexes were only slightly more frequent than RPA-2/RPA-4 (Figure 4G). There was no RPA-1/RPA-2/RPA-4 complex formation in the Pre-Ab and wild type controls (colocalization of Cy3 and Cy5 dyes). However, a few events were noted in the Post-Wash values in all experiments. While unlikely, the appearance of these rare complexes may represent an artifact of the method as it only accounts for less than 1% of the total counts. These complexes may arise from two biotinylated antibodies located within diffraction limited spot on the surface (closer than 250 nm). These signals, however, may also represent actual RPAs whose functions need to be further investigated. Figure 4H highlights all CeRPA complexes we were able to observe. We cannot, however, account for a possibility that formation of these complexes requires additional subunits or post-translational modifications. Existence of the RPA-1/RPA-1 complexes, however, is consistent with the observation that co-depletion of RPA-2 and RPA-4 is viable.

### RPA-2, but not RPA-4, is essential for meiotic recombination

Proper repair of meiotic DSBs is critical to the viability of the resulting gametes. If RPA-2 performs an essential role in meiotic DSB repair, we expect it to be essential for embryonic viability. As expected, deletion of *rpa-2* resulted in reduction in embryonic viability where no eggs hatched in single mutants or *rpa-2; rpa-4* double mutants (Figure 5A). These data suggest that DSB repair *via* HR requires RPA-2. To address this possibility, we examined the diakinesis nucleus adjacent to the spermatheca (diakinesis-1). In wild-type worms, we expect to observe six DAPI bodies in this nucleus, representing the six pairs of homologous chromosomes joined by crossover. Lack of DSB repair will result in DNA fragmentation as each chromosome has several DSBs (31). However, in *rpa-2* hermaphrodites, a range of different DAPI body numbers was observed compared to wild type, indicating that repair of meiotic DSBs occurred (Figure 5B). The irregular size of these DAPI bodies indicates that repair did not use homology and likely involved error-prone DSB repair mechanisms. To test this, we crossed the *rpa-2* mutants to mutants of *cku-70* [part of the canonical non-homologous end joining (cNHEJ) pathway] and/or *polq-1*(part of the alternative end joining pathway). While removing either one of these pathways by itself had no effect on the number of DAPI bodies, an increase was observed in the triple mutant *polq-1; cku-70; rpa-2* strain, confirming the repair of breaks occurs through canonical and alternative end-joining (error-prone) repair mechanisms, and that these pathways act redundantly to repair DSBs in the germline in the absence of *rpa-2* (Supplementary Figure S7A). Thus, while RPA-1 is essential for viability starting from early development (Figure 1B), RPA-2 is essential later, for reproduction.

### RPA-4 localization is upregulated in response to induced DNA damage

Despite the similarity to RPA-2, RPA-4 may not be required under normal growth conditions in which DSBs are programmed (Figure 5A and C). To test whether it is required following exposure to exogenous DNA damage, we introduced DNA damage by IR. This DNA damage is thought to primarily induce DSBs and occurs throughout the germline. Unlike *rpa-2* mutants, *rpa-4* mutant progeny had similar embryonic viability compared to wild type under normal growth conditions (Figure 5A and C). Next, we tested if RPA-4 is required for embryo viability when the germline is challenged with exogenous DNA damage. Following 100 Gy of IR from a cesium source, the viability of *rpa-4* mutant worms and eggs laid was reduced compared to irradiated wild-type control worms, indicating a sensitivity to IR in *rpa-4* worms (Figure 5C and Supplementary Figure S7B and C). These data suggest that RPA-4 plays a significant but smaller role compared to RPA-2 in recombination. When analyzing FLAG::RPA-4 localization following exposure to gamma-IR, RPA-4 localization increased in abundance from <1% to about half of nuclei (Figure 5D and E). To test whether RPA-4 is recruited to other types of exogenous DNA damage, we exposed worms to HU, which creates DSBs by inducing replication stress. RPA-4 localized to transition zone and early pachytene nuclei, with few foci in mitotic zone nuclei, despite HU damage being induced at this stage (Figure 5F). Although the number of RPA-4-positive nuclei increases after exposure to HU (Figure 5F), the level of RPA-1 and RPA-4 colocalization did not significantly change following HU exposure (Supplementary Figure S7D). The presence of RPA-4 in nuclei after exit from mitosis (after DNA damage was processed) is consistent with the observation that RPA-4 is not recruited to DSBs immediately and that it is preferentially localized to pachytene nuclei in unexposed germlines (Figure 3A). To test if the recruitment of RPA-4 to HU breaks effects viability, we tested for the brood size of *rpa-4* mutants following HU exposure. *rpa-4* mutant worms exposed to HU showed a reduction in their brood size following exposure to HU when compared to wild-type unexposed controls (Figure 5G). Altogether these data show that RPA-4 is recruited to DSBs when their levels are increased. The difference in the amount and/or timing of RPA-4 focus formation may reflect a varied response to different types of DNA damage: *rpa-2* mutants (DSBs formed by replication fork collapse and DSB repair defects), MIR (localized clustered DSBs), IR (dispersed DSBs, systemic exposure) or HU exposure (DSBs formed by replication fork collapse).

### RPA-4 inhibits RAD-51 focus formation

RPA is required for efficient RAD-51 recruitment to ssDNA, an essential step in the repair by HR (63–65). Therefore, if RPA loading was impaired, we would not expect RAD-51 to form foci in pachytene nuclei. *rpa-2* deletion severely reduced, but did not eliminate the presence of RAD-51 foci (Figure 6A), confirming a defect in meiotic HR as the source of the abnormal DAPI body phenotype (Figure 5B).

Despite the seemingly wild-type localization of RAD-51 foci in *rpa-4* mutants, the timing of RAD-51 foci appearance was altered. We observed greater numbers of RAD-51 foci in zone 4 and a reduction of RAD-51 foci numbers in zones 6 and 7 in *rpa-4* mutants compared to wild type (Figure 6A). This may indicate more rapid RAD-51 loading and removal in the absence of *rpa-4*, which is consistent with a model by which RPA-4 inhibits RPA-1-mediated RAD-51 recruitment. In agreement, *rpa-2; rpa-4* double mutants had significantly more RAD-51 foci than *rpa-2* single mutants (zones 1, and 5–7). To test this possibility, we analyzed the timing of recruitment of GFP::RAD-51 to MIR foci in the presence and absence of *rpa-4*. This method allows us to create DNA damage that induces DSBs in a timed manner, and thus allows us to calculate the recruitment time of repair proteins to the DNA damage. This analysis was performed as previously described in *spo-11* mutant background to reduce background levels of foci (49). As expected, MIR-induced GFP::RAD-51 foci were significantly more abundant in *rpa-4* mutants (Figure 6B and C), suggesting that RPA-4 indeed attenuates RAD-51 focus formation.

### RPA-4 promotes germline apoptosis in *rpa-2* mutants

In late pachytene about half of meiotic nuclei are eliminated by apoptosis, while surviving nuclei and their chromosomes go through characteristic morphological changes as they transition to diplotene and then diakinesis. These changes are observed in the bend region of the germline, where the gonad that is positioned inside the worm's body bends towards the uterus of the worm, about halfway through the length of each gonadal arm. *rpa-2* and *rpa-4* single mutants exhibited normal progression into diakinesis as found in wild type (Figure 7A). Analysis of the *rpa-2; rpa-4* double mutants uncovered a surprising role for the paralogs in germline progression. Pachytene nuclei were observed extending past the bend in *rpa-2; rpa-4* germlines, where diplotene and diakinesis meiotic stages should occur (Figure 7A). The distance from the bend to the first diakinesis nucleus was slightly increased in *rpa-4* mutants compared to wild type. However, in *rpa-2; rpa-4* mutants this region was ∼6 times longer (∼36 μm compared to 214 μm, Figure 7B). Fewer diakinesis nuclei were observed in *rpa-2* mutants than wild type or *rpa-4* mutants (Figure 7C), which also lead to fewer eggs laid (Supplementary Figure S7E). This was an expected outcome of the reduction in germline proliferation in the *rpa-2* mutant background, leading to reduced numbers of mitotic germline nuclei, and germline length, as shown above. Despite nearly identical effects on germline proliferation (Figure 1E–H), diakinesis nuclei were scarcely found in *rpa-2; rpa-4* double mutants, compared to *rpa-2* mutants (Figure 7C).

In the germline, physiological apoptosis is used as a mechanism for clearing half of the meiotic nuclei so their metabolite content can be supplied to the few oocytes targeted for fertilization (39). Apoptosis is also used for removing damaged cells, but this is not thought to contribute to physiological apoptosis under normal growth conditions. The accumulation of pachytene nuclei in *rpa-2; rpa-4* double mutants could be attributed to defects in apoptosis. To test this hypothesis, we examined the level of apoptosis in the three mutants using a CED-1::GFP reporter. CED-1 is a transmembrane receptor of the *C. elegans* germline that mediates engulfment of apoptotic nuclei (43). While *rpa-4* had no effect on CED-1::GFP engulfment, *rpa-2* mutants exhibited a notable increase in engulfment regardless of whether the total number of apoptotic nuclei was normalized or not to the number of nuclei (Figure 7D and Supplementary Figure S8A). This increase is expected since in the absence of RPA-2, HR is abrogated and nuclei with unrepaired DSBs accumulate. Consistent with RPA-4 playing a role in promoting DNA-damage induced apoptosis, *rpa-2; rpa-4* double mutants displayed wild-type levels of engulfed nuclei in the CED-1 pathway. Apoptotic corpses are removed through three different pathways, and the major germline pathway is CED-1 dependent (66). These pathways all conclude in DNA fragmentation and acidification of the nucleus that can be detected by acridine orange staining (67). Using this approach, we observed that *rpa-2* exhibited increased apoptosis, compared to wild type and *rpa-4* mutant gonads (Figure 7E and F). This increase was in similar level to that observed using the CED-1::GFP analysis (Figure 7D). Apoptosis in *rpa-2* mutants, but not pachytene extension, was dependent on CED-3/Caspase (68,69) (Figure 7G and Supplementary Figure S8B and C). While *rpa-2* mutants have acridine orange staining that was restricted to the bend region where it is normally found in wild-type gonads, in *rpa-2; rpa-4* mutants the majority of acridine orange staining occurred in nuclei of the proximal gonad (Figure 7E and F). Thus, while the CED-1 apoptosis pathway is abrogated in *rpa-2; rpa-4* mutants, apoptosis eventually takes place, although with a notable delay. These data indicate that meiotic progression defects in *rpa-2; rpa-4* double mutants are likely attributed to the combined effect of loss of the CED-1/DNA damage apoptotic response and the delay of apoptosis to the end of prophase I.

In light of the opposing function RPA-4 plays in RAD-51 focus assembly, the genetic interaction between *rpa-2* and *rpa-4* may be interpreted as a requirement of RPA-4 for apoptosis in the presence of persistent DNA damage found in *rpa-2* mutants, related to replication errors. If true, removal of *rpa-4* in a different genetic background that leads to excessive DNA damage specifically in meiotic nuclei (post-replication) should create a phenocopy of the *rpa-2; rpa-4* double mutant. SYP-2 is part of the synaptonemal complex which pairs homologous chromosomes in meiosis I, and mutants of *syp-2* have increased apoptosis due to delay in HR and abrogation of inter-homolog DSB repair. *syp-2; rpa-4* mutants were indistinguishable from *syp-2* single mutants in length of germline and levels and positioning of acridine orange stained nuclei (Supplementary Figure S8D–F). DSB repair defects in *syp-2* mutants are different than those of *rpa-2* mutants, since the former contains ssDNA bound by RAD-51 capable of strand invasion, while the latter does not. To further test our hypothesis, we combined *rpa-4* deletion with *rad-51* mutants that contain resected ssDNA and no functional HR. However, *rpa-4; rad-51* double mutants did not exhibit extension in the length of pachytene/diplotene past the bend and were indistinguishable from *rad-51* mutants in terms of apoptosis levels and timing (Supplementary Figure S8G–I). Taken together, these data indicate that under normal growth conditions, RPA-4 is responsible for an apoptotic signal that is upstream of RAD-51 binding, and likely responds to DNA damage due to replication errors found in *rpa-2* mutants.

Next, we aimed to identify where RPA-4 acts in licensing the apoptotic pathway. Mitogen-activated protein kinase (MAPK) has been identified as an important factor influencing germline apoptosis, and mutants of *mpk-1* show germline arrest prior to diplotene (45,70). We stained for activated MAPK (di-phosphorylated MPK-1) and found that all *rpa-2; rpa-4* double mutant gonads displayed normal MAPK staining, as found in wild type (Supplementary Figure S9). This indicates that normal ‘licensing’ for apoptosis is occurring in *rpa-2; rpa-4* double mutants, but commitment to apoptosis is altered. These data suggest that RPA-4 acts downstream of MAPK.

### RPA-4 acts to promote germline apoptosis in aging germlines and following DNA damage

RPA-4 is not required for apoptosis under normal growth conditions in young adult worms (Figure 7D–F). However, RPA-4 is found in low levels under these conditions and is only recruited to DNA following exogenous DNA damage. It is therefore possible that RPA-4 promotes apoptosis in challenging conditions. Germline apoptosis has been shown to increase in aging worms (71). Therefore, we examined the localization of *rpa-4* foci in 3-day-old adults and found a significant increase in RPA-4 foci numbers compared to 1-day-old adults (Figure 8A). Unlike what is found in young adults, RPA-4 foci numbers were partially dependent on SPO-11-induced breaks as they were reduced in the *spo-11* mutants. If the elevation of RPA-4 foci in aged worms was important for apoptosis, then apoptosis in these worms will be *rpa-4* dependent. Indeed, apoptosis levels in 3-day-old adults were reduced in *rpa-4* mutants compared to wild type (Figure 8B). An effect on oocyte numbers was also observed following exposure to IR and was mildly suppressed in *rpa-4* mutants (Figure 8C).

When engulfed nuclei of wild-type germlines were examined for co-occurrence with RPA-4, they showed a small increased preference for RPA-4 (Figure 8D). The small increase can be explained by the positioning of RPA-4-positive nuclei. RPA-4 disappeared (12.8 ± 6.1 nuclear rows before diplotene) when CED-1-positive nuclei first appeared with overlap of only 3–4 rows (the first 23% of the apoptosis zone). These data altogether indicate that RPA-4 plays a role in apoptosis under challenging conditions, marking nuclei destined for apoptosis, but disappearing as the apoptotic program progresses.

## DISCUSSION

These data provide insight into the roles of alternative RPA complexes (RPA-1/RPA-2 and RPA-1/RPA-4) in *C. elegans* meiosis (summarized in Supplementary Figure S10A). We demonstrated that RPA (RPA-1/2) is essential for replication and recombination as is seen in other organisms, and we identified an additional role of RPA (RPA-1/4) in regulating germline development. This work offers the first look at the role of RPA-2 and RPA-4 in the *C. elegans* germline, as well as a comprehensive investigation of RPA-1. Our data suggest that RPA-1 and RPA-2 are essential for normal germline replication, and that RPA-4 acts as part of a quality control mechanism promoting germline apoptosis. Our work suggests that RPA-4 evolved to provide a response for conditions in which DSB repair is impaired (older worms) or challenged by excessive damage (IR/HU). Despite the similarity between RPA-4 and RPA-2, these proteins evolved to play different and antagonistic functions in DSB repair (Supplementary Figure S10B).

### RPA-1 and RPA-2, but not RPA-4, are critical for normal germline replication and HR repair

Previous reports have demonstrated that the large, medium, and small RPA subunits are essential for replication and recombination across organisms (16). We examined the function of the known RPA subunits in *C. elegans*: one RPA1 and two RPA2-like subunits. Our studies suggested that no trimeric complex is formed between these known subunits. This does not exclude the presence of a third, yet unidentified subunit that performs a function similar to RPA3. This is formally possible as canonical metazoan RPA is a trimeric complex (72). Instead, all pairwise combinations are possible. While RPA-1/2 and RPA-1/4 may be expected, based on studies in other organisms, the RPA-2/4 dimer is unexpected and may explain the genetic interactions between RPA-2 and RPA-4 (see below). The presence of homodimers, is also puzzling and it is not clear yet whether these are formed on ssDNA.

Our data are consistent with a role for *C. elegans* RPA-1 and RPA-2 in replication (likely as RPA-1/2 complex). However, while RPA-1 is essential for replication, RPA-2 is not. This suggests that RPA-1 can bind ssDNA and facilitate replication without the need to bind RPA-2. The ability of *C. elegans* RPA-1 to homodimerize may explain this observation. Instead, RPA-1 activity may be enhanced by RPA-2 binding. Our localization data are consistent with this model, as RPA-1 can localize to mitotic germline nuclei in the absence of *rpa-2*, albeit in lower levels than wild type. This observation is perplexing, as it was assumed that in metazoans RPA is an obligatory trimeric complex. In other organisms it was shown that RPA3 is recruited to the RPA complex by physically interacting with RPA1 and RPA2. The ability of RPA-1 to promote replication by itself may also explain why a putative RPA-3 has not yet been described in *C. elegans* or is possibly missing. Indeed, despite the presence of several OB fold containing proteins in *C. elegans* (wormbase) none of them were annotated as RPA proteins. RPA proteins need to contain not only an OB fold, but also the essential aromatic amino acids within it (73). Despite the sequence similarities, we have seen no evidence that RPA-4, the RPA-2 ortholog, plays a role in replication. *rpa-4* mutants show no phenotypes related to a replication defect and RPA-4 does not extensively localize to proliferative zone nuclei even under HU stress.

The second conserved function of the RPA complex is in promoting HR repair by binding to ssDNA following resection, which is essential for RAD-51 recruitment. Our data are consistent with RPA-1 and RPA-2 playing a conserved role in this process. Unlike what is found for replication, RPA-2 plays an essential role in HR. This may be a result of an interaction of RPA-2 with resection factors, or with proteins that allow for loading of RAD-51 (31,62). In its absence HR fails and despite the ability of RPA-1 to localize to the ssDNA formed, it cannot support efficient RAD-51 recruitment. In *rpa-2* mutants the DSBs formed in meiosis are repaired by cNHEJ and TMEJ, while HR is abrogated. These data suggest that the RPA-1-ssDNA filament (that does not contain RPA-2) is more permissive to replication than to HR. As with replication, RPA-4 does not seem to play a role in HR under normal conditions.

### RPA-4 acts in promoting DNA-damage induced apoptosis in challenging conditions

Under normal growth conditions (young adults with no exogenously induced damage), deletion of *rpa-4* does not effect any phenotype indicative of a function in DNA damage repair. The localization pattern of RPA-4 in these conditions indicates that very few nuclei require RPA-4’s function, and that RPA-4’s foci are not localized to SPO-11-induced breaks. However, under challenging conditions (older worms/gamma-IR/HU/*rpa-2* mutants), RPA-4 is found in more nuclei and is promoting apoptosis. We interpret these data as indicating a role of RPA-4 in targeting a subset of nuclei, ones with unrepairable DSBs, to apoptosis.

Our results place RPA-4 as part of the apoptotic pathway, downstream of MAPK signaling. The fact that RPA-4 foci are found in pachytene, and only in the most distal engulfed nuclei and not just in late pachytene argues that RPA-4 acts prior to the implementation of apoptosis (which is restricted to late pachytene). RPA-4 is recruited to DNA damage foci following RPA-1. We propose that following DNA damage RPA-1/2 localizes to DSBs. While some DSBs can be promptly repaired, others may be more challenging to repair. In normal conditions these may include DSBs generated by replication errors, but in aging worms altered DNA repair processes may also channel some SPO-11-induced DSBs to this pathway. The identified nuclei are then marked by RPA-4, and these nuclei move to late pachytene, where MAPK signaling occurs and apoptosis is executed, concurrently with the removal of RPA-4.

### RPA-2 and RPA-4 are important for normal meiotic progression

We show that RPA-2 is essential for normal germline repair, while RPA-4 is important for DNA-damage induced apoptosis. In *rpa-2; rpa-4* double mutants, apoptotic nuclei are observed in the proximal gonad. In these double mutants, wild-type levels of CED-1::GFP engulfment are observed, indicating that physiological apoptosis is not disturbed. This also indicates that RPA-4 is required for the DNA damage-related increase in CED-1::GFP engulfment in *rpa-2* mutants. Undoubtedly, the majority of damaged nuclei are allowed to escape apoptosis at the bend region, unable to transition into diplotene/diakinesis nuclei and get eliminated aberrantly at the end of meiotic prophase I, by a CED-1-independent engulfment pathway. In *daz-1* mutants, pachytene-to-diplotene transition is defective, and results in a similar phenotype that we observe in our *rpa-2; rpa-4* double mutants where apoptotic nuclei are observed in the proximal gonad (74). Therefore, we believe that the loss of this transition in *rpa-2; rpa-4* double mutants may be due in part to the excessive DNA damage resulting from HR and normal replication abrogation, leading to defective transition signaling.

### RPA-4 and RPA-2 function antagonistically in regulating RPA-1 focus formation.

RPA-2 performs a canonical function in DSB repair by promoting RPA-1 activity. In its absence, RPA-1 focus formation is impaired, but the effect is much larger on RAD-51 focus formation, indicating that in addition to facilitating RPA-1 engagement with DNA, RPA-1/2 complex can support RAD-51 focus formation more efficiently than RPA-1 by itself. RPA-2 not only promotes RPA-1 function but also inhibits RPA-4 focus formation. This may be an indirect action due to RPA-1/2 being a complex that more easily associates with ssDNA than RPA-1, outcompeting RPA-4 for binding. Alternatively, RPA-2 can effect RPA-4’s ability to bind ssDNA. Indeed, in our single-molecule experiments we have shown that RPA-2 binds RPA-4 forming a stable complex. This interaction may negatively and directly regulate RPA-4 binding to ssDNA.

Despite high similarity between RPA-2 and RPA-4, RPA-4 not only plays a completely different function in HR, but also an antagonistic role to RPA-2. Instead of promoting RAD-51 focus assembly, RPA-4 attenuates it. Our MIR studies show that RPA-4 is recruited to DSBs following RPA-1. We propose that RPA-4 recruitment to RPA-1 foci can disassociate RPA-1 to an extent that ssDNA does not contain enough RPA-1 to support RAD-51 loading, or RPA-4 prevents the displacement of RPA-1 by RAD-51. As a result, the nucleus is committed to apoptosis. Since RPA-4 recruitment is delayed, this can provide enough ‘buffer time’ for the nucleus to load RAD-51 and repair the DSB through HR. Repair of challenging DNA damage is delayed, resulting in RPA-4 recruitment and apoptosis.

## DATA AVAILABILITY

Strains are available upon request. The authors state that all data necessary for confirming the conclusions presented in the article are represented fully within the article.

## Supplementary Material

gkaa1293_Supplemental_Files
