# Correction to ‘RPA complexes in Caenorhabditis elegans meiosis; unique roles in replication, meiotic recombination and apoptosis’

**DOI:** 10.1093/nar/gkab631

**Published:** 2021-08-09

**Authors:** Adam Hefel, Masayoshi Honda, Nicholas Cronin, Kailey Harrell, Pooja Patel, Maria Spies, Sarit Smolikove

**Affiliations:** Department of Biology, The University of Iowa, Iowa City, IA 52242, USA; Department of Biochemistry, The University of Iowa Carver College of Medicine, Iowa City, IA 52242, USA; Department of Biochemistry, The University of Iowa Carver College of Medicine, Iowa City, IA 52242, USA; Department of Biology, The University of Iowa, Iowa City, IA 52242, USA; Department of Biology, The University of Iowa, Iowa City, IA 52242, USA; Department of Biochemistry, The University of Iowa Carver College of Medicine, Iowa City, IA 52242, USA; Department of Biology, The University of Iowa, Iowa City, IA 52242, USA

The authors wish to introduce the following corrections to their article (1).

1) The DNA sequence for the *OLLAS::RPA-1* is incorrect. The correct sequence is:

TTCCCCAATTTTTATGTATCTGTTTCAGATAGTGAAAGATGTCCGGATTCGCCAACGAGCTCGGACCACGTCTCATGGGAAAGGCGGCAATTCACATCAATCACGATGTCTTCAATAA

2) In some places in the list of Strains *rpa-1* was indicated to be placed on chromosome I, when its correct position is on chromosome II.

The published article has been updated. These changes do not affect the results, discussion and conclusions presented in the article.
