# Supplementary material for: RPA complexes in Caenorhabditis elegans meiosis; unique roles in replication, meiotic recombination and apoptosis
Source: Nucleic Acids Res. 2021 Jan 21;49(4):2005–26. doi: 10.1093/nar/gkaa1293 (PMC7913698; doi:10.1093/nar/gkaa1293)
Supplement: gkaa1293_Supplemental_Files [file gkaa1293_supplemental_files.zip › Hefel 2020- Sup-R2.pdf]

RPA complexes in *Caenorhabditis elegans* meiosis; unique roles in replication, meiotic recombination and apoptosis

Adam Hefel[1], Masayoshi Honda[2+], Nicholas Cronin[2+], Kailey Harrell**[1], Pooja Patel**[1], Maria Spies[2] and Sarit Smolikove[1,*]

[1] Department of Biology, The University of Iowa, Iowa City, Iowa, 52242, USA

[2] Department of Biochemistry, The University of Iowa Carver College of Medicine, Iowa City, Iowa, 52242, USA

Supplementary Figures and their Sample size

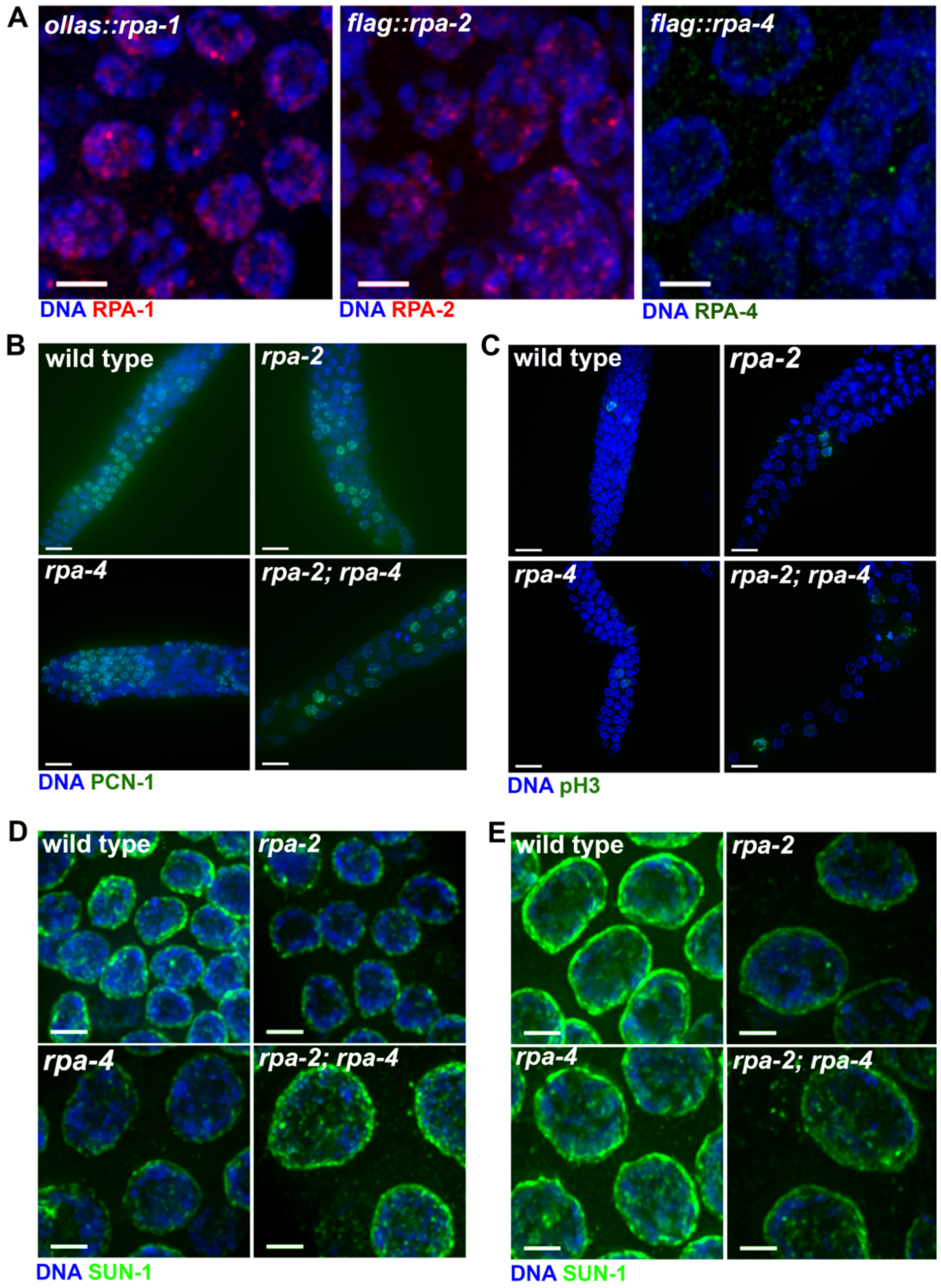

Figure S1: example IF staining for Figure 1.

A) Examples of tagged RPA subunit staining in PMT nuclei used for analysis in Figure 1C. Scale bar is 2μm. B) Examples of PCN-1 staining in PMT nuclei used for analysis in Figure 1E. Scale bar is 10μm. C) Examples of pH3 staining in PMT nuclei used for analysis in Figure 1F. Scale bar is 10μm. D) Examples of SUN-1 staining in PMT nuclei used for analysis in Figure 1H. Scale bar is 2μm. E) Examples of SUN-1 staining in MP nuclei used for analysis in Figure S5B. Scale bar is 2μm.

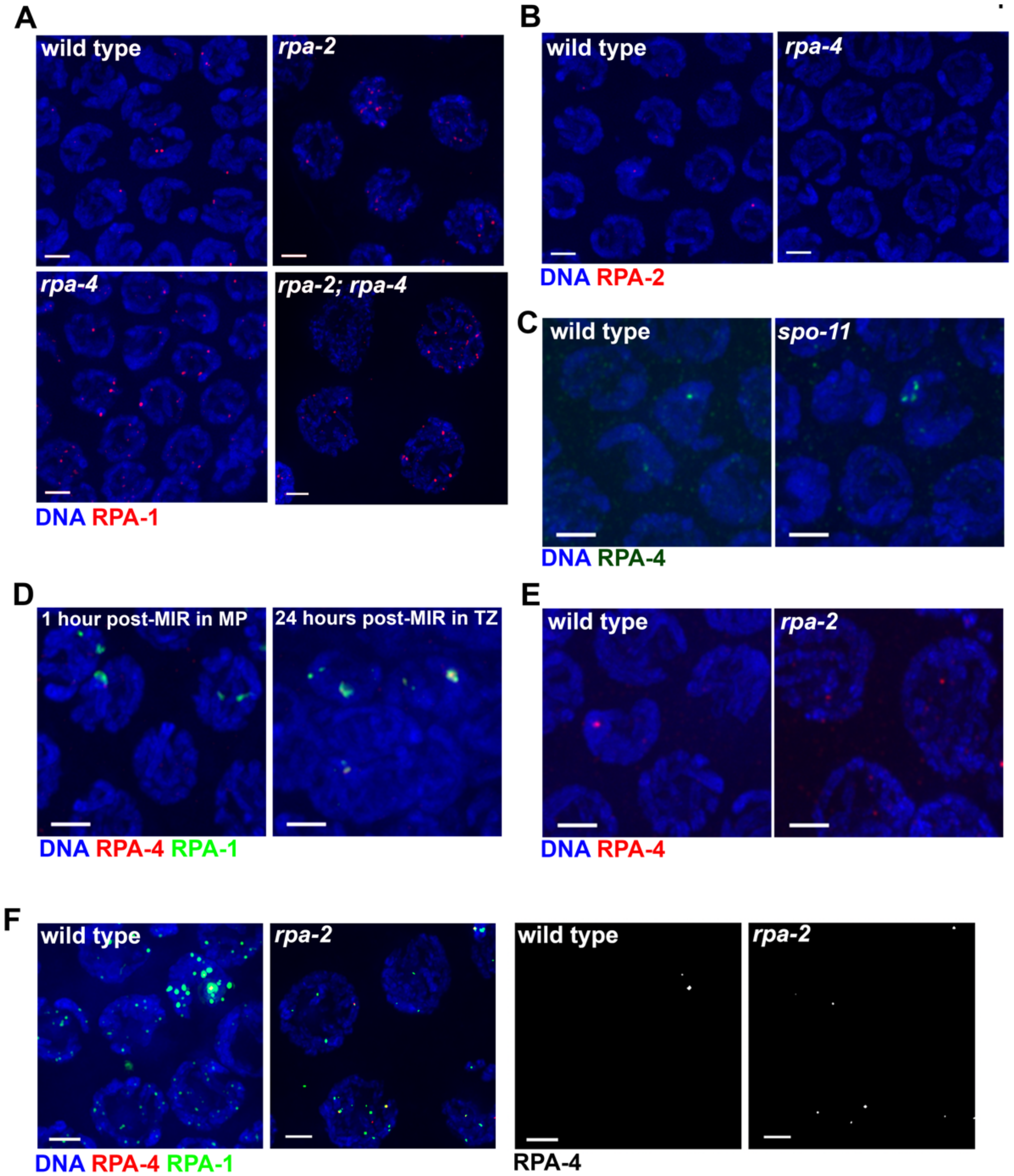

Figure S2: example IF staining for Figures 2-3.

A) Examples of OLLAS::RPA-1 staining in pachytene nuclei used for analysis in Figure 2A. Scale bar is 2µm. B) Examples of FLAG::RPA-2 staining in pachytene nuclei used for analysis in Figure 2B. Scale bar is 2µm. C) Examples of FLAG::RPA-4 staining in pachytene nuclei used for analysis in Figure 3A. Scale bar is 2µm. D) Examples of OLLAS::RPA-1 and FLAG::RPA-4 co-staining in pachytene nuclei used for analysis in Figure 3B. Scale bar is 2µm. E) Examples of FLAG::RPA-4 staining in pachytene nuclei used for analysis in Figure 3C. Scale bar is 2µm. F) Examples of OLLAS::RPA-1 and FLAG::RPA-4 co-staining in pachytene nuclei used for analysis in Figures 3GH. Scale bar is 2µm.

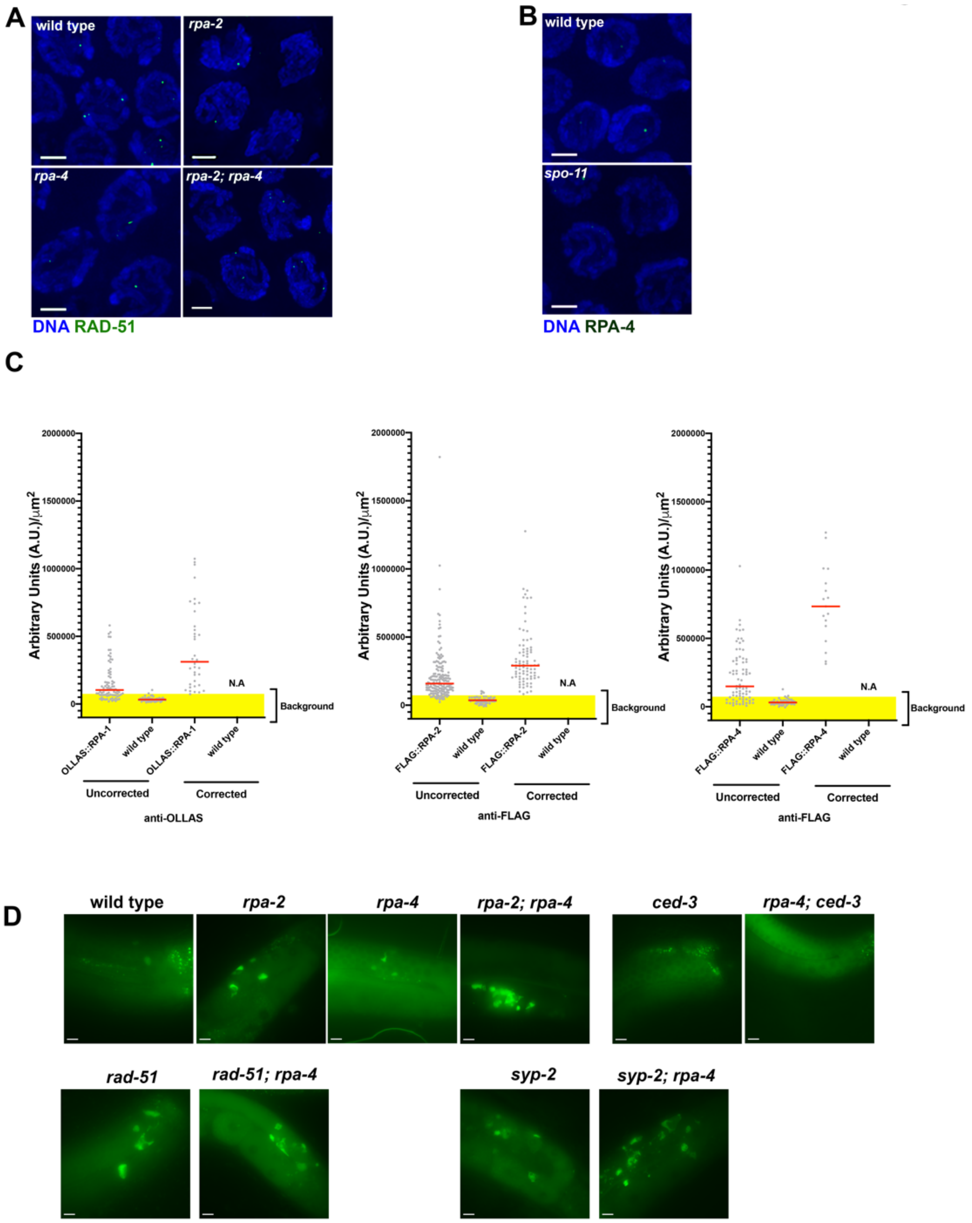

Figure S3: example IF staining for Figures 4-8 and background intensity correction examples.

A) Examples of RAD-51 staining in pachytene nuclei used for analysis in Figure 6A. Scale bar is 2μm. B) Examples of FLAG::RPA-4 staining in 3-day-old pachytene nuclei used for analysis in Figure 8A. Scale bar is 2μm. C) Intensity of OLLAS::RPA-1 (left), FLAG::RPA-2 (middle), and FLAG::RPA-4 foci used in Figures 2A, 2B, and 3C respectively before and after background correction where correction was done visually by eliminating all background cytoplasmic foci before measuring intensity. Yellow bar represents the intensity of foci recorded in wild type (no tag) staining controls. D) Examples of acridine orange staining. Scale bar is 10μm.

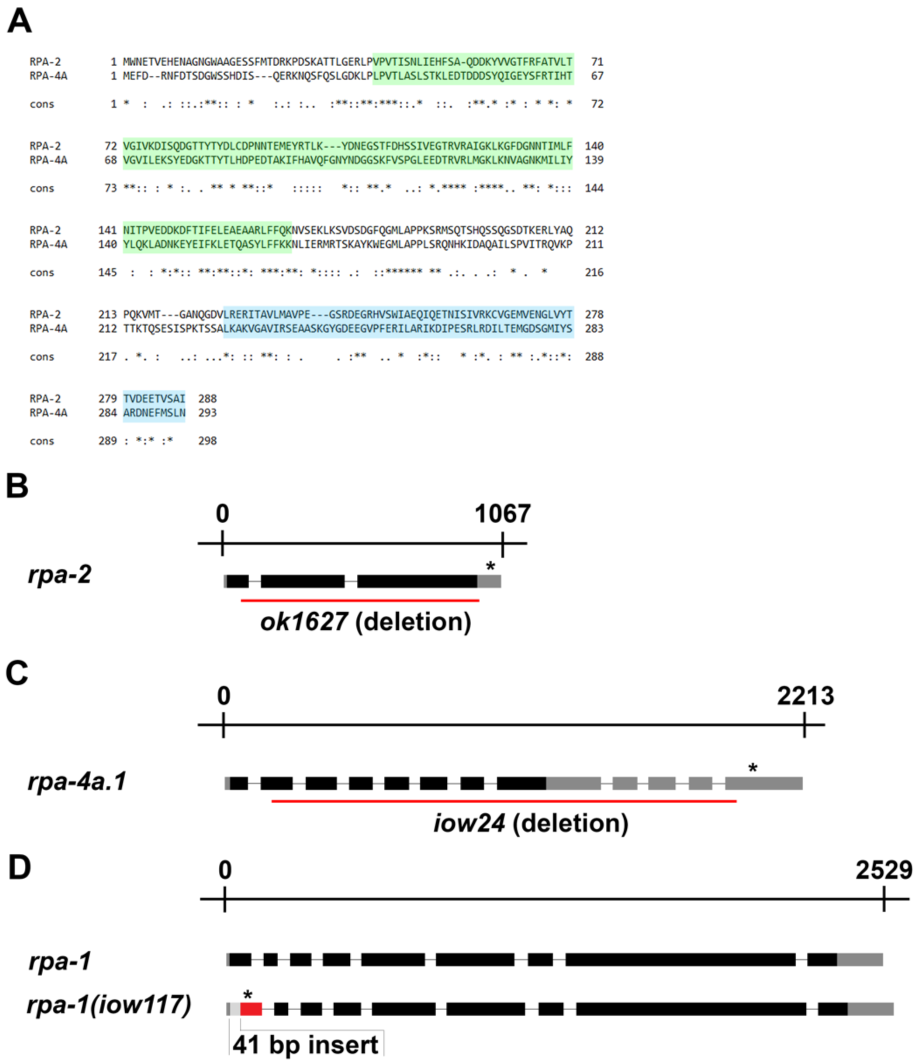

Figure S4: Alignment of RPA-2 and RPA-4 protein sequences, and mutant illustrations.
A) ClustalW alignment of RPA-2 with RPA-4. Green box indicates OB-fold domain. Blue box indicates winged-helix domain. B-D) Illustrations of the corresponding genes and mutations used in this manuscript. Black lines represent introns. Black boxes represent exons. Grey boxes represent untranslated regions. Asterisk represents premature stop codon.

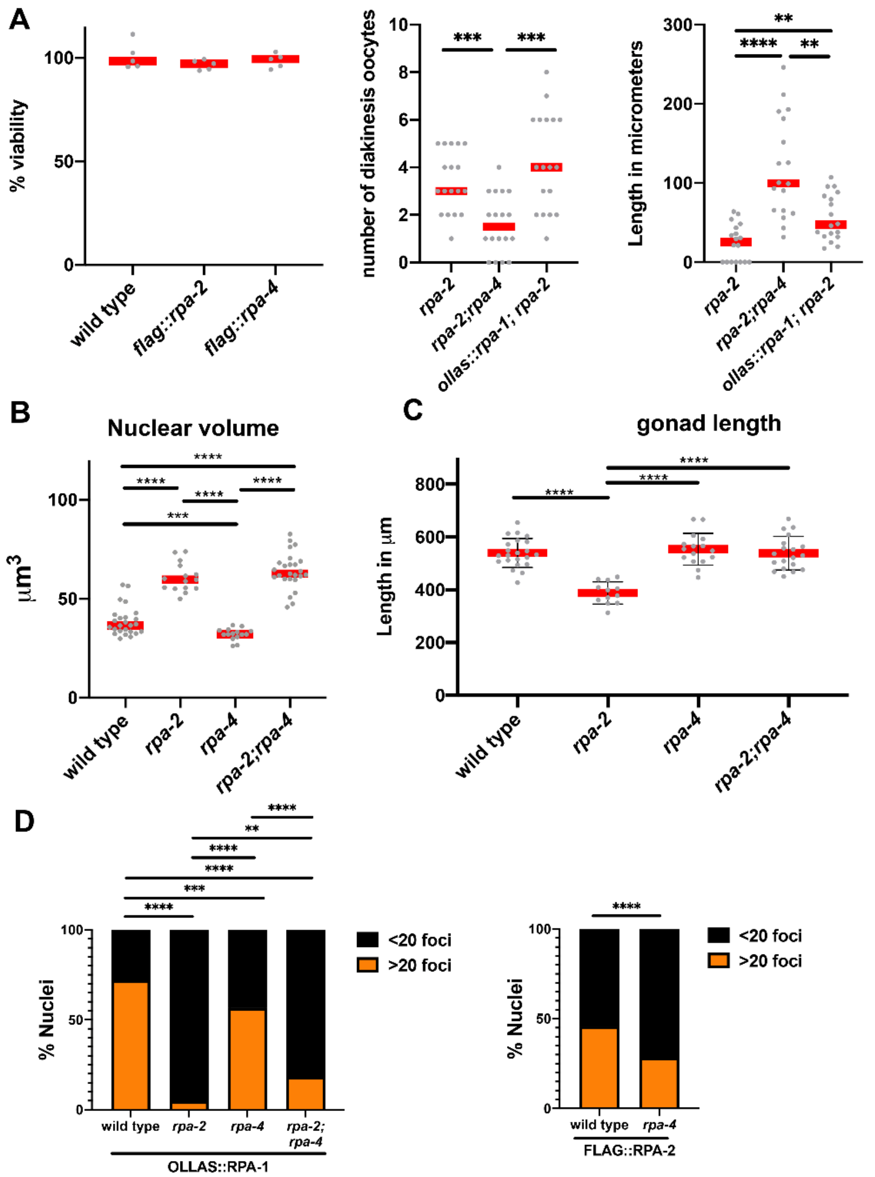

Figure S5: Viability of tags, nuclear volume of MP nuclei, gonad length, and percent of nuclei with >20 foci of indicated stain.

A) Percent of viable hatched eggs(left), number of diakinesis oocytes(middle), and length of extended pachytene(right) in tagged mutants compared to wild type and rpa-2; rpa-4 double mutants. B) Mid-pachytene nuclear volumes as estimated by calculations using FIJI acquired data of SUN-1 stained nuclei(see materials and methods). C) Lengths of entire gonads from Carnoy's fixed worms. D) Quantification of the number of nuclei with >20 foci for OLLAS::RPA-1 staining (left) and FLAG::RPA-2 staining (right). Mann-Whitney tests performed for panels A, B, and D and T-test performed with welches correction for C, where p-values are represented as ****=<0.0001, ***=<0.001, **=<0.01, and * =<0.05. Red lines in A, B, and D indicate the median and in C the mean with standard deviation.

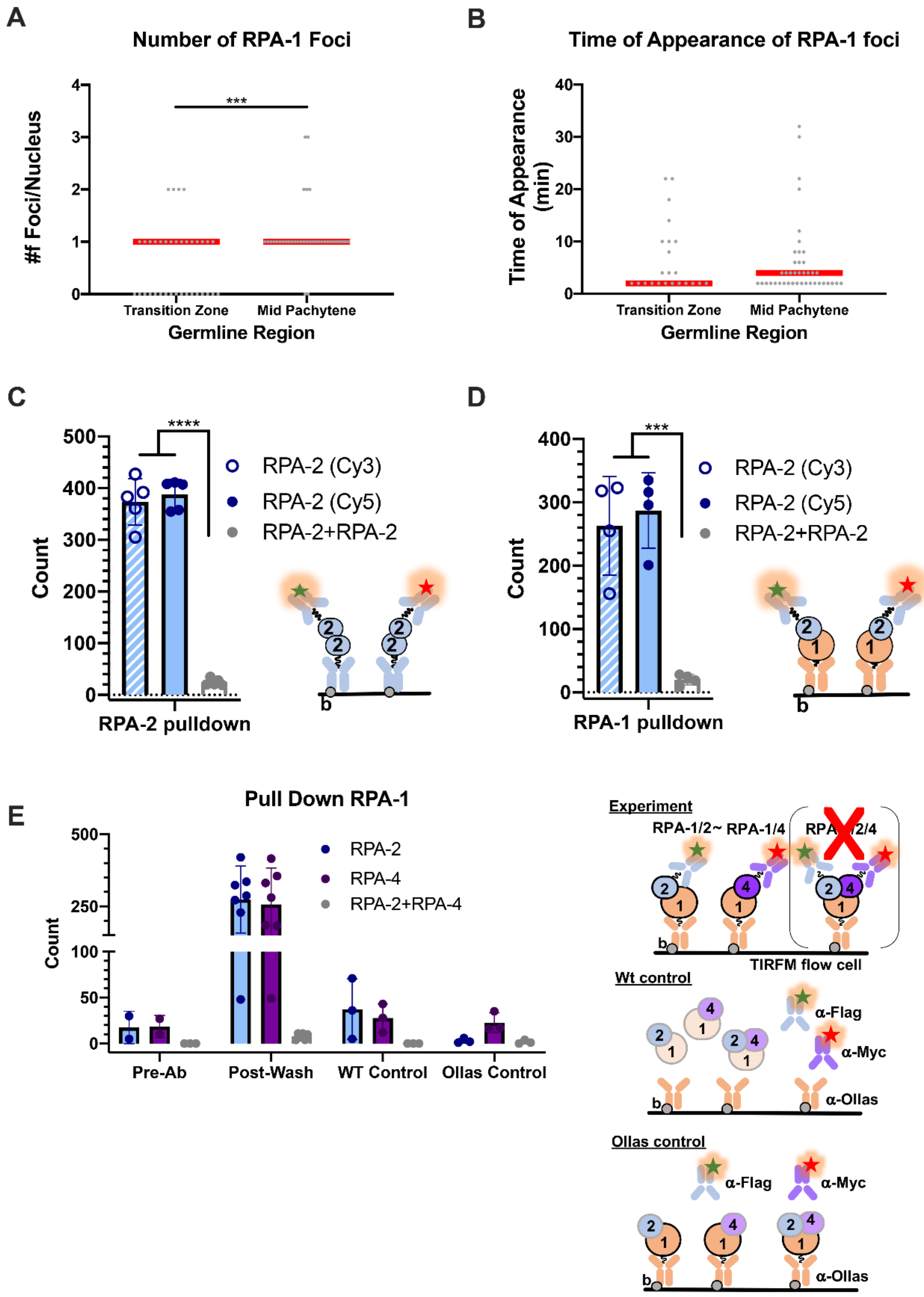

Figure S6: GFP11::RPA-1 focus formation and controls for SiMPull experiments

A) Number of GFP11::RPA-1 foci following laser MIR in TZ and MP nuclei. B) Time of appearance for GFP11::RPA-1 foci following laser MIR. Mann-whitney tests performed. Red lines indicate the median. C) A control experiment showing that we are observing homo-dimers and not higher

oligomeric species. Here, the RPA complexes we pulled to the TIRFM flow-cell surface using surface-tethered biotinylated anti-FLAG antibodies (RPA-2) and the RPA complexes were visualized and quantified with Cy3- and Cy5-labeled anti-FLAG antibodies. The absence of co-localization for the Cy3 and Cy5 signal suggest the absence of the trimeric or higher oligomeric species of RPA-2. D) Control, showing that RPA-1 and RPA-2 form a dimeric complex. No RPA-1/2x-RPA-2 complexes were observed. Panels C and D also show that labeling the same antibody with Cy3 or Cy5 die does not affect its tag recognition. E) As shown in Figure 4B, the pull-down experiment was carried out with the biotinylated antibodies against a tag on one of the RPA subunits (OLLAS tag here) tethered to the surface of the TIRFM flow cell. Pre-Ab control shows non-specific binding of the Cy3-labeled anti-FLAG (RPA-2) and Cy5-labeled anti-MYC (RPA-4) in the absence of the surface-tethered anti-OLLAS. The lysate from animal with OLLAS::RPA-1, FLAG::RPA-2 and MYC::RPA-4 was used in this control. Therefore, the events observed in this control combine non-specific binding of the antibodies to the surface and specific binding of the antibodies to RPA molecules non-specifically bound to the surface. Post-Wash is the actual experiment (see Materials and Methods for detail). Wt-Control used lysate from wild-type worms expressing untagged RPAs. Ollas-Control used lysate from worms expressing OLLAS::RPA-1 and untagged RPA-2 and RPA-4. Cartoon representations of the experiment and two controls are depicted on the right of the graph.

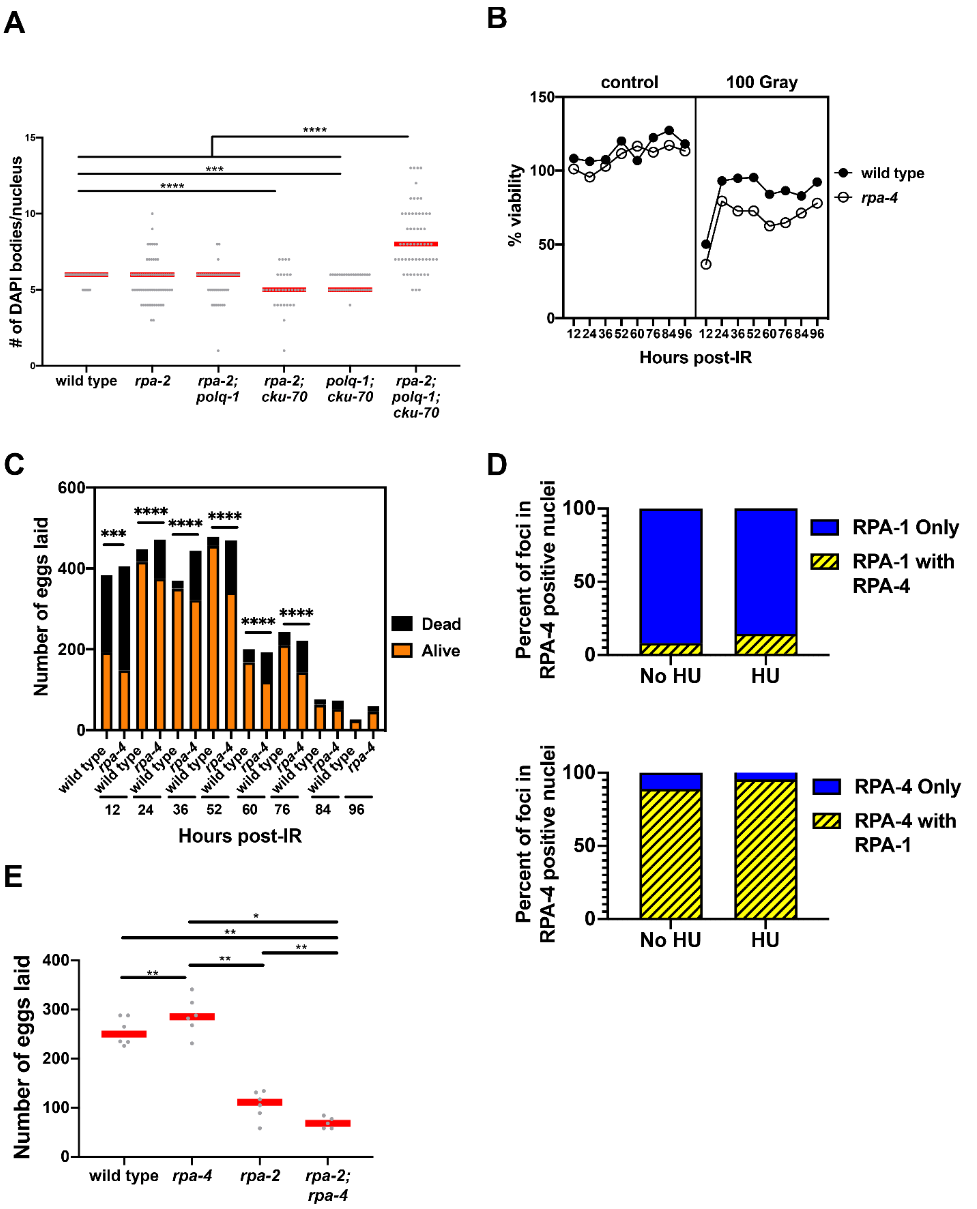

Figure S7: DAPI body counts for various DSB repair mutants, colocalization of RPA-1 and RPA-4 foci in HU treated worms, viability after gamma IR, and number of eggs laid by each mutant.

A) Number of DAPI bodies counted in diakinesis -1 nuclei for indicated DSB repair mutants. B) Percent of viable eggs at indicated time points in gamma irradiated and control worms. C) Dead and alive eggs per hour time period indicated on the x axis compared between wild type and *rpa-4* mutants. D) Percent of OLLAS::RPA-1 and FLAG::RPA-4 focus colocalization in HU treated and control worms.. E) Number of eggs laid by each mutant genotype. Mann-Whitney tests performed where p-values are represented as ****=<0.0001, ***=<0.001, **=<0.01, and * =<0.05. Red lines indicate the median.

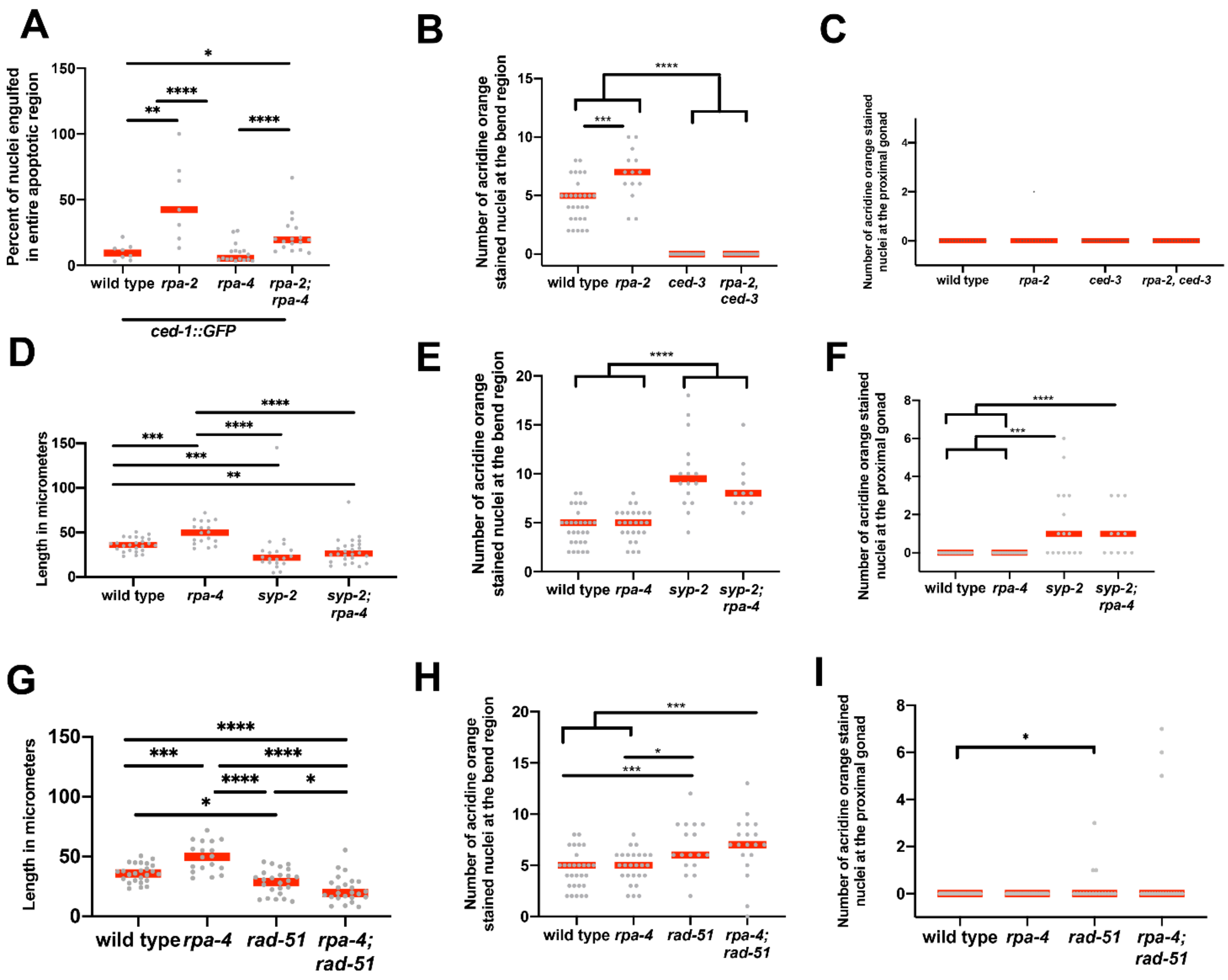

Figure S8: Apoptosis analysis using CED-1::GFP engulfment, pachytene extension, and acridine orange staining.

A) Number of CED-1::GFP engulfed nuclei in each indicated genotype. B, E, and H) Number of Acridine Orange staining nuclei in distal gonad measured until the end of the “bend” region. C, F, and I) Number of Acridine Orange staining nuclei in proximal gonad measured from the end of the “bend” region to the spermatheca. D and G) Length of pachytene extension in indicated mutants measured from bend to first diakinesis nucleus. Mann-Whitney tests performed where p-values are represented as ****=<0.0001, ***=<0.001, **=<0.01, and * =<0.05. Red lines indicate the median.

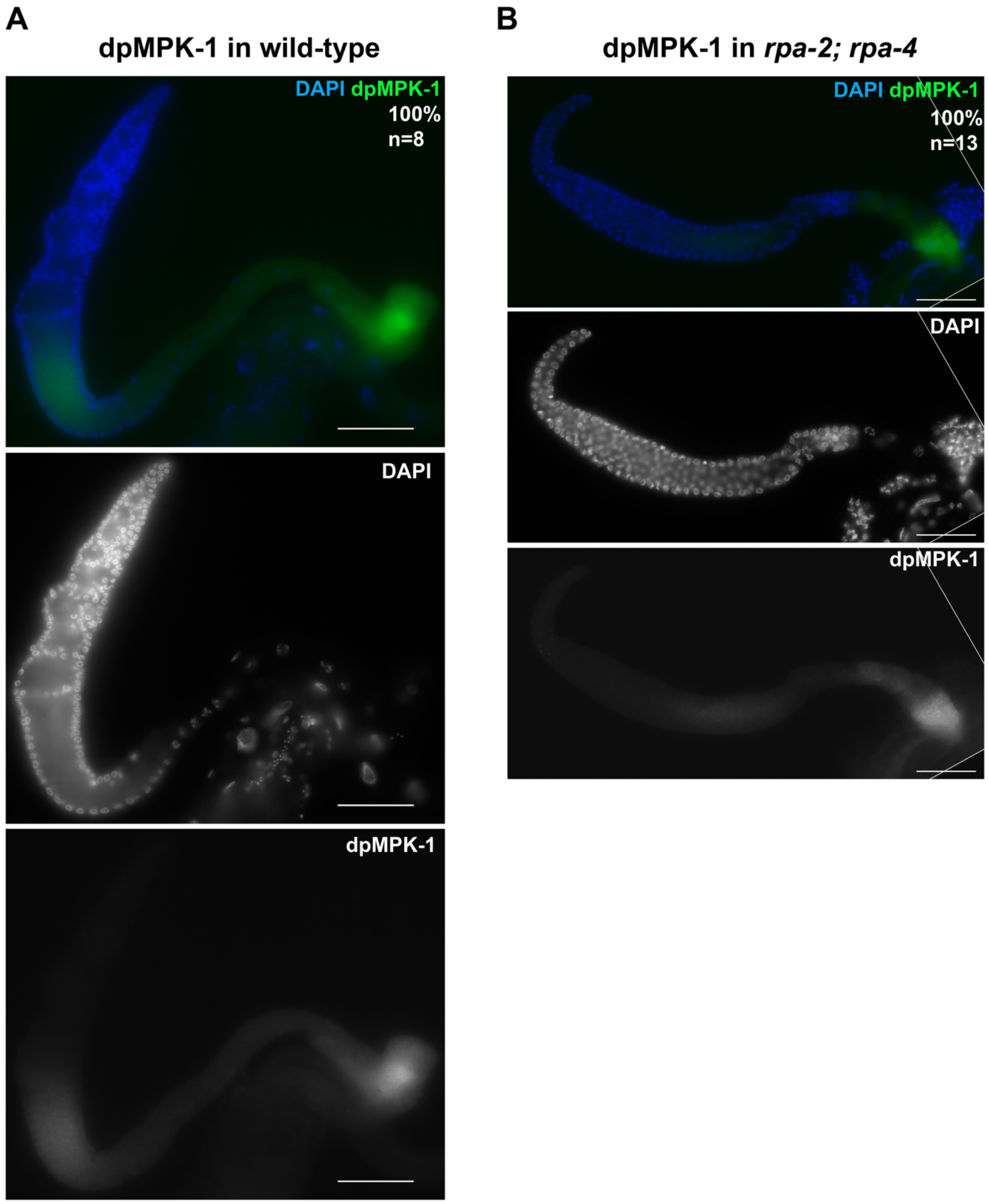

Figure S9: di-phosphorylated MPK-1 staining representative images of wild type and *rpa-2;rpa-4* gonads.

Gonads dissected and stained with dpMPK-1. All *rpa-2; rpa-4* double mutant gonads displayed normal MAPK staining (n=13), as found in wild-type (n=8).

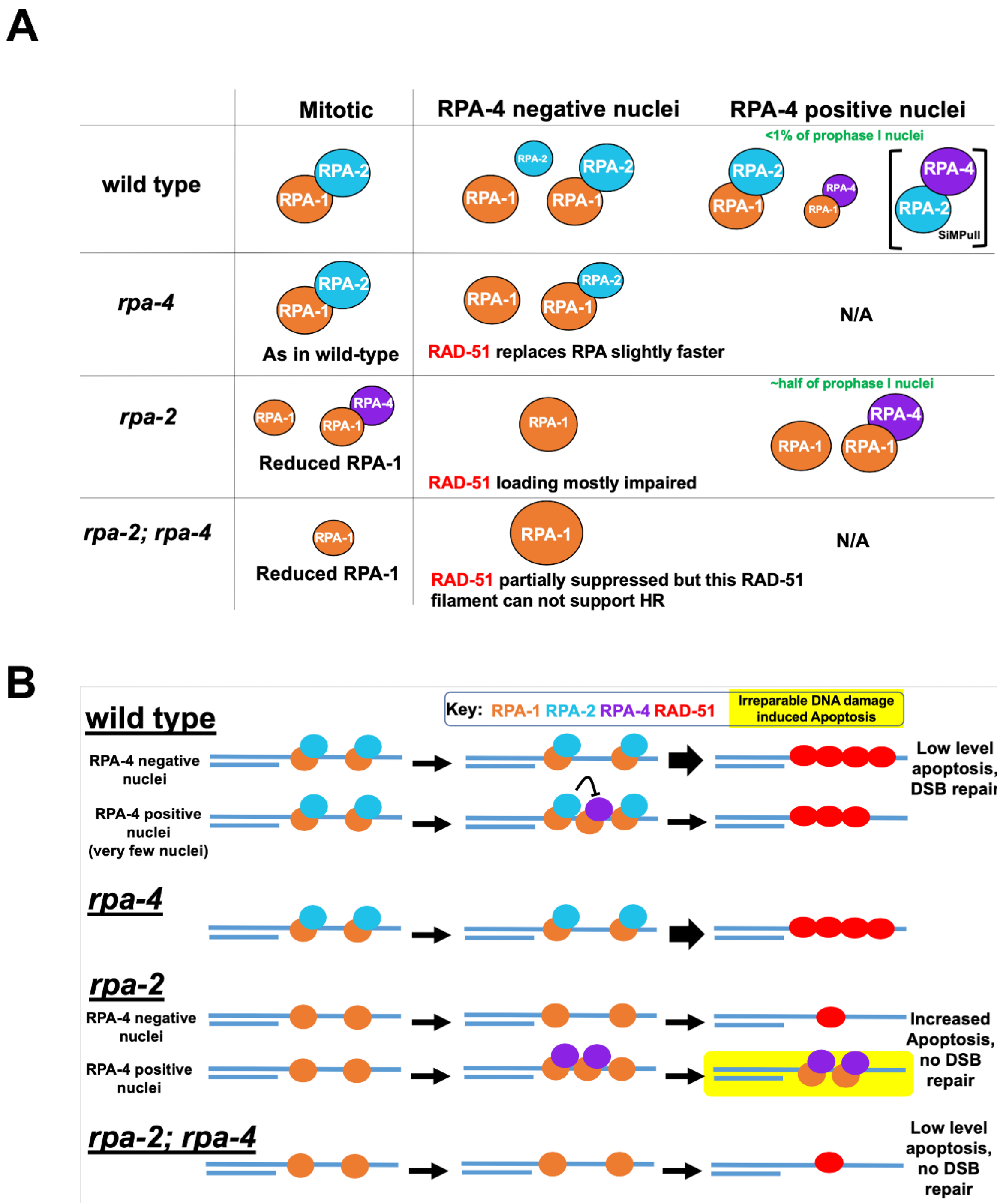

Figure S10: Models for RPA complexes and their effects on RAD-51 loading and apoptosis

A) Pictographic description of the cytological and SiMPull data in each genotype, B) Model in context of ssDNA binding, RAD-51 loading, and apoptosis.

Sample size for Supplementary Figures

Figure S3C: uncorrected OLLAS::RPA-1 n=86, uncorrected wild type n=42, corrected OLLAS::RPA-1 n=32. uncorrected FLAG::RPA-2 n=173, uncorrected wild type n=58, corrected FLAG::RPA-2 n=84. uncorrected FLAG::RPA-4 n=77, uncorrected wild type n=46, corrected FLAG::RPA-4 n=17.

Figure S5: the left panel A shows analysis of n=5 P0s for each genotype, the middle and right panel A shows n=18 gonads analyzed for each genotype, panel B shows analysis of n gonads/nuclei for wild type n=5/25, *rpa-2* n=3/15, *rpa-4* n=3/15, and *rpa-2; rpa-4* n=5/25, panel C shows analysis of n gonads for wild type n=22, *rpa-2* n=12, *rpa-4* n=16, and *rpa-2; rpa-4* n=19, the left panel D shows analysis of n nuclei for *ollas::rpa-1* n=232, *ollas::rpa-1;rpa-2* n=85, *ollas::rpa-1;rpa-4* n=242, and *ollas::rpa-1;rpa-2; rpa-4* n=120, and the right panel D shows the analysis of n nuclei for *flag::rpa-2* n=329 and *flag::rpa-2; rpa-4* n=304.

Figure S6: panel A shows the analysis of n nuclei for TZ and MP n=36, panel B shows the analysis of n foci for TZ n=23 and MP n=41, panel C shows analysis of n=5 experiments with n pulldown counts for RPA-2/RPA-2(Cy3) n=1868, for RPA-2/RPA-2(Cy5) n=1939, and for RPA-2/RPA-2(Cy3)/RPA-2(Cy5) n=123, panel D shows analysis n=3 experiments of n pulldown counts for RPA-1/RPA-2(Cy3) n=797, for RPA-1/RPA-2(Cy5) n=852, and for RPA-1/RPA-2(Cy3)/RPA-2(Cy5) n=62, panel E shows analysis of n=2/7/3/3 experiments for pre-ab control/Post-wash/WT control/OLLAS-RPA-1 with n pulldown counts for RPA-1/RPA-2 n=35/1916/112/9, for RPA-1/RPA-4 n=37/1810/83/68, and for RPA-1/RPA-2/RPA-4 n=0/51/0/5.

Figure S7 panel A shows the analysis of n diakinesis - 1 nuclei for wild type n=56, *rpa-2* n=65, for *rpa-2; polq-1* n=44, *rpa-2; ku-70* n=32, *polq-1; ku-70* for n=38, and *rpa-2; polq1; ku-70* n=58, top panel B shows analysis of n foci for no HU n=98 for and HU treated n=882, bottom panel B shows analysis of n foci for no HU treatment n=9 and HU treated n=134, panels C shows analysis of n P0s for wild type control n=7, *rpa-4* control n=6 , wild type IR n=9 and *rpa-4* IR n=8, panel D shows analysis of n P0s for wild type IR treated n=9 and *rpa-4* IR treated n=9, panel E shows analysis of n=6 P0s for each genotype.

Figure S8 Panel A shows analysis of n gonads for *ced-1::gfp* n=8, *ced-1::gfp; rpa-2* n=7, *ced-1::gfp; rpa-4* n=17 and *ced-1::gfp; rpa-2; rpa-4* n=17, panel B and C shows analysis of n gonads for wild type n=29, *rpa-2* n=14, *ced-3* n=22, and *rpa-2; ced-3* n=17, panel D shows analysis of n gonads for wild type n=25, *rpa-4* n=18, *syp-2* n=20, and *syp-2; rpa-4* n=26, panels E and F shows analysis of n gonads for wild type n=29, *rpa-4* n=26, *syp-2* n=11 and, *syp-2; rpa-4* n=16, panel G shows analysis of n gonads for wild type n=25, *rpa-4* n=18 for, *rpa-4;rad-51* n=24 for, and *rad-51* n=24, panels H-I shows analysis of n gonads for wild type n=29, *rpa-4* n=26, *rpa-4;rad-51* n=19 for, and *rad-51* n=17.

For more information about sample size refer to Sup table.
